# MecA in *Streptococcus mutans* is a multi-functional protein

**DOI:** 10.1128/msphere.00308-24

**Published:** 2024-11-12

**Authors:** Kassapa Ellepola, Robert C. Shields, Jessica K. Kajfasz, Hua Zhang, Jose A. Lemos, Hui Wu, Zezhang T. Wen

**Affiliations:** 1 Department of Oral and Craniofacial Biology, School of Dentistry, Louisiana State University Health Sciences Center, New Orleans, Louisiana, USA; 2 Department of Biological Sciences, Arkansas State University, Jonesboro, Arkansas, USA; 3 Department of Oral Biology, College of Dentistry, University of Florida, Gainesville, Florida, USA; 4 Department of Restorative Dentistry, School of Dentistry, Oregon Health and Science University, Portland, Oregon, USA; 5 Department of Microbiology, Immunology and Parasitology, School of Medicine, Louisiana State University Health Sciences Center, New Orleans, Louisiana, USA; The University of Arizona, Tucson, Arizona, USA

**Keywords:** *Streptococcus mutans*, *mecA*, *clpP*, biofilms, dental caries, adaptor proteins

## Abstract

Our recent studies have shown that deficiency of MecA in *Streptococcus mutans* significantly affects cell division, growth, and biofilm formation. In this study, an *in vitro* mixed-species model, proteomics, and affinity pull-down assays were used to further characterize the MecA-mediated regulation in *S. mutans*. The results showed that compared with the wild type, UA159, the *mecA* mutant significantly reduced its production of glucans and weakened its ability to facilitate mixed-species biofilm formation. Relative to the wild type, the *mecA* mutant also displayed unique characteristics, including colony morphology, growth rate, and biofilm formation that did not fully resemble any of the *clpP, clpX, clpE, clpCE,* and *clpC* individual or combinational mutants. Deletion of *mecA* was shown to result in alteration of >337 proteins, including down expression of GtfBC&D and adhesin P1. More than 277 proteins were differentially expressed in response to *clpP* deletion, including increased expression of GtfB. By cross-referencing the two proteomes, a distinctive set of proteins was found to be altered in the *mecA* mutant, indicating a ClpP-independent role of MecA in the regulation of *S. mutans*. When analyzed using affinity pull-down, ClpC, ClpX, ClpE, and CcpA were among the members identified in the MecA-associated complex. Further analysis using a bacterial two-hybrid system confirmed CcpA, ClpX, and ClpE as members of the MecA interactome. These results further suggest that MecA in *S. mutans* is more than an adapter of the Clp-proteolytic machinery, although the mechanism that underlies the Clp-independent regulation and its impact on *S. mutans* pathophysiology await further investigation.

**IMPORTANCE:**

MecA is known as an adaptor protein that works in concerto with ATPase ClpC and protease ClpP in the regulated proteolysis machinery. The results presented here provide further evidence that MecA in *S. mutans*, a keystone cariogenic bacterium, plays a significant role in its ability to facilitate mixed-species biofilm formation, a trait critical to its cariogenicity. Proteomics analysis, along with affinity pull-down and bacterial two-hybrid system, further confirm that MecA can also regulate *S. mutans* physiology and biofilm formation through pathways independent of the Clp proteolytic machinery, although how it functions independently of Clp awaits further investigation.

## INTRODUCTION

*Streptococcus mutans* is a primary inhabitant in plaque biofilms and isconsidered a major etiological agent of human dental caries. Fermentation of dietary sugars by *S. mutans* leads to acid production that causes demineralization of the tooth enamel. *S. mutans* possesses multiple mechanisms to colonize and form biofilms on tooth surfaces, metabolize a wide range of carbohydrates, and tolerate rapid and frequent environmental fluctuations such as pH, oxidative stress, and osmolarity, characteristics that are important for the growth, persistence, and survival (1). *S. mutans’* unique cellular biology and biofilm forming ability that allow it to thrive in the oral cavity are significantly influenced by a unique combination of virulence attributes including multiple two component signal transduction systems, molecular chaperones, quorum sensing, and biofilm regulatory proteins such as BrpA and autolysin AtlA, high affinity adhesin P1 (a.k.a. SpaP), glucan binding proteins (Gbps), and glucosyltransferase enzymes (GtfBC&D) (2–4).

*S. mutans* biofilm formation is also regulated in response to various environmental cues and the presence of other oral bacterial species. Constant exposure to dietary sucrose favors the production of extracellular polysaccharides (EPS, a.k.a. glucans) by *S. mutans* via Gtf’s and enhances adhesive interactions between *S. mutans* and other organisms, especially the cariogenic microorganisms that establish and accumulate within the EPS-rich matrix while providing structural integrity to the biofilm and an increase of the acidogenicity of the biofilm matrix (5, 6). Among the odontopathic bacteria populating the carious dentine, studies assessing the predominant cultivable flora in this complex ecosystem have identified *Streptococcus oralis*, *Actinomyces naeslundii*, *Lactobacillus casei*, and *Veilonella* spp. along with *S. mutans* (7, 8).

As a consequence of the exposure of *S. mutans* cells to these environmental stresses, the accumulation of abnormal proteins can increase due to errors in transcription and translation that need to be essentially countered through the maintenance of protein homeostasis (9–11). The Clp proteolytic complex is important for bacterial viability, persistence, growth, and maintenance of protein homeostasis, which is achieved through the stabilization of proteins that play essential functions and degradation of mis-folded or aberrant proteins and functions under normal growth conditions as well as under stress conditions (12–14). The Clp proteolytic complex of *S. mutans* comprised of AAA^+^ ATPase subunits such as ClpE, ClpX, or ClpC that help recognize and translocate the protein substrates into the proteolytic core of a proteolytic component known as ClpP peptidase, where subsequent proteolysis takes place (13–15).

Adapter proteins such as MecA and SspB play an important role in modulating the function of the Clp proteolytic machinery. MecA is an adaptor protein widespread among Gram-positive bacteria required for functional ClpC complex formation (16–19). The activated, MecA-ClpC molecular complex harnesses the energy of ATP binding and translocates the unfolded polypeptide to the ClpCP degradation machine (20). It was also shown that ClpC, only together with MecA, can disaggregate and refold aggregated proteins in *Bacillus subtilis* (18).

MecA-mediated proteolysis has also been shown to play a role in the regulation of competence development in *Bacillus* spp. and oral streptococci. In streptococci, MecA acts as a negative regulator on alternative sigma factor SigX, also known as ComX, which is a transcriptional activator of late competence genes (21–23). MecA forms the SigX-MecA-ClpC complex that helps ClpP recognize and degrade SigX, thus modulating the expression of genes required for the development of genetic competence (21, 24, 25). In *S. mutans* deficiency of MecA, ClpC or ClpP was shown to result in cellular accumulation of SigX and a prolonged competence state, although over expression of MecA enhances proteolysis of SigX and accelerates the escape from competence (26, 27).

Our recent studies have generated evidence that in addition to the role of an adapter in the ClpC/P proteolytic machinery, MecA in *S. mutans* may also possess functions that are independent of the ClpP protease (28). Like MecA, *clpP* deletion also led to reduced biofilms when growing in glucose, but unlike MecA, the *clpP* mutant increased biofilm formation in the presence of sucrose (29). Consistently, ClpP deficiency led to >4-fold increases in GtfB, C, and D expressions (14), whereas *mecA* mutant displayed a major reduction in GtfB and GtfC (28). In this study, we further examined the effects of *mecA* deletion on *S. mutans* biofilm formation in a mixed-species consortium and used proteomics, affinity pull down and bacterial two-hybrid system to explore if and how MecA functions independently of the Clp machinery. The results presented here provided further evidence that MecA-deficiency in *S. mutans* significantly affects its ability to facilitate mixed-species biofilm formation and leads to phenotypes that none of the *clp* mutants fully resemble. In addition, proteomics analysis also showed that major differences exist in the altered protein profiles between the *mecA* and *clpP* mutant. Affinity pull-down and bacterial two-hybrid system analysis revealed that ClpE, ClpX, and CcpA among others are MecA-interactive proteins, although if these proteins are Clp independent awaits further investigation.

## MATERIALS AND METHODS

### Bacterial strains and culture conditions

All bacterial strains used in this study are listed in Table 1. *S*. *mutans* wild-type UA159 and its derivatives were maintained in brain heart infusion (BHI) with addition of erythromycin (Erm, 10 µg/mL), spectinomycin (Spe, 1 mg/mL), and/or kanamycin (Kan, 1 mg/mL) to the growth medium, when necessary. *Streptococcus oralis* and *A. naeslundii* were also maintained in the BHI medium. *L. casei* was maintained in Lactobacillus MRS (Difco Laboratories, Detroit, MI) broth. For growth studies, the wild type and its derivatives were grown in BHI broth to an optical density at 600 nm (OD_600_) of 0.5; the cultures were diluted 1:100 in fresh BHI broth, and the growth at 37°C was assessed using a Bioscreen C (30). For mixed-species biofilm formation, ultra-filtered tryptone yeast extract (UYTYE) medium supplemented with 18 mM glucose and 2 mM sucrose was used (31). Solid media were prepared by adding 1.5% (wt/vol) Bacto agar (Difco Laboratories, Detroit, MI). Unless otherwise stated, cultures were maintained in an aerobic chamber at 37°C with inclusion of 5% CO_2_.

**TABLE 1:** Bacterial strains and plasmids used in this study^*a*^

Strain or plasmid	Relevant characteristics	Reference(s)
*S. mutans* UA159	Wild-type ATCC	ATCC
*S. mutans* TW416	UA159/∆*mecA*, Spc^r^	De et al*.* (28)
*S. mutans* TW416fs	UA159/*mecA,* markerless frameshift mutation	This study
*S. mutans* TW416C	UA159/∆*mecA/gtfA::PmecA*, Spc^r^ Kan^r^	(28, 32)
*S. mutans* dCas9	UA159/∆*cas9_smu_/PxlR::dcas9,* Kan^r^	Shields et al*.* (33)
*S. mutans* mecAi	dCas9/pPM-sgRNA-*mecA*, Kan^r^, Spc^r^	This study
*S. mutans* TW471	UA159/∆*clpP,* Erm^r^	This study
*S. mutans* clpPi	dCas9/pPM-sgRNA-*clpP*, Kan^r^, Spc^r^	This study
*S. mutans* clpX	UA159/∆*clpX*, Kan^r^	Kajfasz et al. (29)
*S. mutans* clpCE	UA159/∆*clpCE*, Spc^r^, Kan^r^	Kajfasz et al. (29)
*S. mutans* clpC	UA159/∆*clpC,* Spc^r^	Kajfasz et al*.* (29)
*S. mutans* clpE	UA159/∆*clpE,* Kan^r^	Kajfasz et al*.* (29)
*S. oralis*	SK34	Lab stock
*A. naeslundii*	ATCC 12104	Lab stock
*L. casei*	4646	Lab stock
*E. coli* BACH101	*cya-99*	BACTH kit, Euromedex
*E. coli* DMH1	*cya-854*, *recA1*	BACTH kit, Euromedex
*E. coli* DH10B	Cloning host, *mcrA, mcrBC, mrr,* and *hsd*	Invitrogen, Inc.
pFW5-luc	Integration vector, Spc^r^	Kreth et al*.* (34)
pQE30	Expression vector, Kan^r^	Qiagen, Inc.
pPM-sgRNA	Integration vector for cloning of sgRNA, Kan^r^	Shields et al. (33)
pUT18	Encodes amino acids 225 to 399 of CyaA, Amp^r^	BACTH kit, Euromedex
pUT18C	Encodes amino acids 225 to 399 of CyaA, Amp^r^	BACTH kit, Euromedex
pKT25	Encodes the first 224 amino acids of CyaA, Kan^r^	BACTH kit, Euromedex
pKNT25	Encodes the first 224 amino acids of CyaA, Kan^r^	BACTH kit, Euromedex
pUT18C-zip	pUT18C fused with the leucine zipper of GCN4	BACTH kit, Euromedex
pKT25-zip	pKT25 fused with the leucine zipper of GCN4	BACTH kit, Euromedex

^*a*^ Note: Kan^r^, Erm^r^, Spc^r^, and Amp^r^ indicate kanamycin, erythromycin, spectinomycin, and ampicillin resistance, respectively.

### Construction of markerless and conditional mutants

To eliminate any potential polar effects of the antibiotic resistance marker on the flanking regions, a markerless nucleotide deletion and frameshift *mecA* mutant was constructed by double crossover homologous recombination. Briefly, a 5.0 kb fragment flanking nucleotide adenine at position 30 (A30) and with deletion of A30 (30∆A) of the *mecA* coding sequence were amplified by recombinant PCR using Q5 DNA polymerase (New England Biolabs) (Table S1), and the resulting amplicon was used to transform *S. mutans* UA159 (ATCC 700610) as described by Junges et al. (35). The transformants were plated on a BHI agar plate, and the resulting 30∆A frameshift mutants were screened and verified by Sanger sequencing. To construct a conditional *mecA* and *clpP* mutant, the CRISPRi system was employed as described by Shields et al. (33). Briefly, a sequence of 20 nucleotides (nt) targeting nt 96 to 119 of the *mecA* messenger RNA was selected as single-guide RNA and inserted into the guide RNA plasmid pPM-sgRNA using a Q5 mutagenesis kit (New England Biolabs). Following sequence verification by Sanger sequencing, the resulting construct, pPM-sgRNA-mecA, was transformed into *S. mutans* dCas9_smu_, which carries a markerless *cas9* deletion complemented with a *dcas9* gene fused to an inducible *xylR* promoter in shuttle vector pDL279. The transformants were plated on BHI agar plates with Kan and Spe. For construction of a conditional *clpP* mutant, a sequence of 20 nt targeting nt 13 to 32 of *clpP* messenger RNA was cloned in pPM-sg-RNA and transformed into dCas9_smu_ similarly as described above.

### Mixed-species biofilm formation and microscopy

*S. mutans* UA159 and the *mecA* mutant, tagged with green fluorescent protein (GFP, Ex 395 nm/Em 509 nm) (28, 30), *A. naeslundii*, *S. oralis,* and *L. casei,* were grown till mid-log phase in their respective medium as described above. The cultures were centrifuged, and the cell pellet washed once with phosphate buffered saline (PBS) (20 mM, pH 7.0) was adjusted to OD_600_of ~0.5 in PBS. The cultures were diluted 1:100 in UFTYE medium supplemented with 18 mM glucose and 2 mM sucrose as the supplemental carbohydrate sources, and biofilms were allowed to grow on polystyrene 96-well plates (Corning, New York) or hydroxylapatite discs (HA) vertically deposited in 24-well plates as previously described (36, 37). By the end of 24 and 48 hours, biofilms on 96-well plates were stained using 0.1% crystal violet and measured using a synergy II plate reader (BioTek) at 575 nm (36). For confocal microscopy analysis, 24 h, 48 h, and 5 days mixed-species biofilms on HA disks were stained at the end of incubation with FM 1–43 dye (Ex 473 nm/Em 579 nm) (FisherScientific) that stains the membrane lipids with red fluorescence. Glucan produced was labeled by inclusion of Alexa-Fluor 647 conjugated dextran (1 mM, final conc) (Ex 594 nm/Em 633 nm) (FisherScientific). Biofilms were dissected using a confocal laser scanning microscope (CLSM, Olympus, Japan). Post-acquisition analysis, such as biofilm thickness, biovolume was analyzed using COMSTAT to determine the total biofilm and biofilms of *S. mutans* (UA159 and MecA deficient mutant) and the other bacterial strains, respectively (38).

### Protein expression, affinity chromatography, and protein identification

For MecA expression, the *mecA* coding sequence was amplified by PCR using high fidelity Q5 DNA polymerase and directly cloned in pQE30 by BamHI/SalI fusion. Following sequence verification, the resulting construct was transformed into *E. coli* M15, *mecA* expression was induced using isopropyl β-D-1-thiogalactopyranoside (IPTG, 0.5 mM), and recombinant MecA (rMecA) was purified by affinity chromatography using Ni-NTA resins (Qiagen, Inc.). For affinity pull-down (39) of MecA-interactive proteins, mid-exponential phase (OD_600_ = 0.4) *S. mutans* cultures were harvested by centrifugation, and the resulting cell pellets were resuspended in ice-cold KMNP buffer (2 mM MgCl_2_, 50 mM NaCl, 50 mM K_2_HPO_4_/KH_2_PO_4_, PMSF). The cells were mechanically disrupted using a glass bead beater. Ni-NTA resins were used to immobilize the His-tagged rMecA (bait protein) and incubated with the *S. mutans* cells lysate overnight at 4°C. The protein mixture was introduced into a chromatography column and following multiple washing steps to remove unbound protein, MecA, and its associated complexes were eluted under native conditions. Following SDS-PAGE analysis, the interacting proteins were identified using liquid chromatography mass spectrometer (LC/MS) at LSUHSC Proteomics Center.

### Bacterial two-hybrid (B2H) assays

B2H analysis was carried out by following the protocol of the BACTH system kit (Euromedex) (40, 41). The BACTH system is based on the interaction-mediated reconstitution of the two catalytic domains of *Bordetella pertussis* adenylate cyclase (Cya) in *E. coli*. Briefly, genes of interest were PCR amplified and directly cloned into fusion vectors pUT18C and pKT25 (Table S1). After Sanger sequencing verification, the resulting pUT18C- and pKT25-derivatives were co-transformed into *E. coli* BTH101 simultaneously, and the co-transformants were selected from LB plus 100 µg/mL ampicillin and 50 µg/mL kanamycin. For testing of interaction, 5 µL of each interaction strain was washed in PBS and then plated onto LB agar plus 5-bromo-4-chloro-3-indolyl-β-D-galactopyranoside (X-gal, 50 µg/mL) and isopropyl β-d-1-thiogalactopyranoside (IPTG) (0.5 mM). T18-zip and T25 Zip were used as a positive control, as recommended by the manufacturer (Euromedex).

### Proteomics and western blot analysis

For proteomics, *S. mutans* UA159 and its *mecA* and *clpP* mutants were grown in BHI until mid-exponential phase (OD_600_ of ~0.3), and bacterial cells were homologized using a glass bead beater as described (30). Whole lysates (100 µg of total protein) were digested with trypsin, labeled using a Tandem Mass Tag (TMT) 10-plex Reagent set (Fisher Scientific, Pierce), and then further analyzed using LC/MS as previously described (42, 43). A total of four technical repeats were conducted for each sample.

Western blot analysis was used to verify the expression of selected proteins using whole cell lysates described above. Briefly, 10 µg of total proteins were separated by 7.5% SDS-PAGE, blotted onto a polyvinylidene fluoride (PVDF) membrane, probed using protein-specific polyclonal (for beta-exo-fructosidase FruA and carbon catabolite repressor protein CcpA, kind gift of Dr. R. A. Burne) or monoclonal (for GtfB, a kind gift of the late Dr. W. H. Bowen, and P1, a kind gift of Dr. L. J. Brady) antibodies, and the signals were developed using a SuperSignal West Pico Chemiluminescent kit (Fisher Scientific) (44).

### Statistical analysis

Quantitative data were analyzed using paired Student *t* test. A *P* value of 0.05 or less is considered statistically significant.

## RESULTS

### The MecA-deficient mutants display characteristics distinctive from the Clp mutants

**Fig 1:**
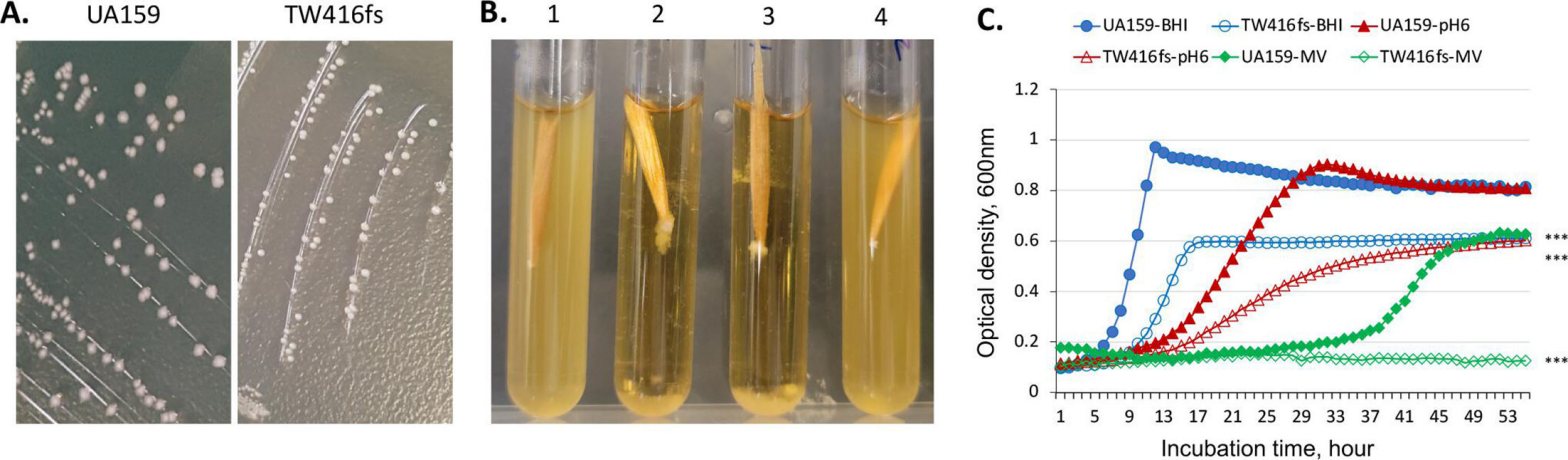
Growth phenotypes of the MecA mutant. (**A**) Colony morphology of the markerless frameshift *mecA* mutant (TW416fs) and its parent (UA159), when grown on BHI agar plates. (**B**) Growth of the wild type (UA159, 1), its *mecA* mutant (TW416fs, 2&3), and the complement strain (TW416fsc, 4) when grown in BHI. (**C**) Growth curves of the *mecA* mutant (TW416fs) and its parent strain (UA159) in Bioscreen C when grown in regular BHI, BHI with pH adjusted to 6.0, and BHI plus methyl viologen at 12.5 mM. ***, *P* < 0.001 in growth rate and optical density of the mutant vs UA159 under the same conditions via Student's *t* test.

Our previous study showed that *mecA* and its downstream *rgpG,* encoding the first enzyme of the rhamnose-glucose polymer (RGP) biosynthesis pathway (45), can be co-transcribed as an operon. To ensure that the phenotypes of the allelic exchange *mecA* mutants are not a result of polar effects on the downstream *rgpG,* a markerless *mecA* mutant was constructed by double crossover homologous recombination of the chromosomal *mecA* with a frameshift mutant copy that was generated by recombinant PCR with 30∆A of the *mecA-*coding sequence. Like the allelic exchange *mecA* mutant, TW416, the markerless frameshift mutant, TW416fs, also formed wet colonies with a shining smooth surface on agar plates (Fig. 1A), severely aggregated in broth medium (Fig. 1B) and displayed major growth defects including a reduced growth rate and a reduced overnight culture density, especially when grown at low pH and in the presence of oxidative stressors (Fig. 1C; Table S2). Like the allelic exchange mutant, the frameshift mutant also had an extended log phase when growing in BHI medium adjusted to pH 6.0 and especially when growing in the presence of an oxidative stressor, methyl viologen (Fig. 1C). These results, along with the fact that a wild-type copy of *mecA* plus its cognate promoter was able to fully complement the allelic exchange mutant (28), confirm that the phenotypes of the *mecA* mutants result from the MecA-deficiency, but not because of any polar effects.

A knock-down *mecA* mutant*,* TW416i was generated using a CRISPR interference system. This CRISPRi mutant has the single-guide RNA constitutively expressed in the dead Cas9_smu_ strain, which expresses Cas9_smu_ when xylose at 0.1% (wt/vol) is included in the growth medium. Like TW416, albeit to a lesser degree, the knockdown mutant, TW416i, also formed clumps and mostly settled in the bottom of the test tubes when grown in chemically defined medium FMC with the addition of xylose (0.1%, wt/vol) (Fig. S1A), but such phenotypes were not observed when xylose was absent from the culture medium. When grown in a Bioscreen C with shaking, TW416i also displayed a reduced growth rate when xylose was included in the culture medium, although the optical density (OD_600_) of the overnight cultures was just slightly reduced when compared with those without xylose (Fig. S1B). These results also validate the significant role of *mecA* in *S. mutans* physiology.

### MecA deficiency in *S. mutans* affects its ability to form mixed-species biofilms

**Fig 2:**
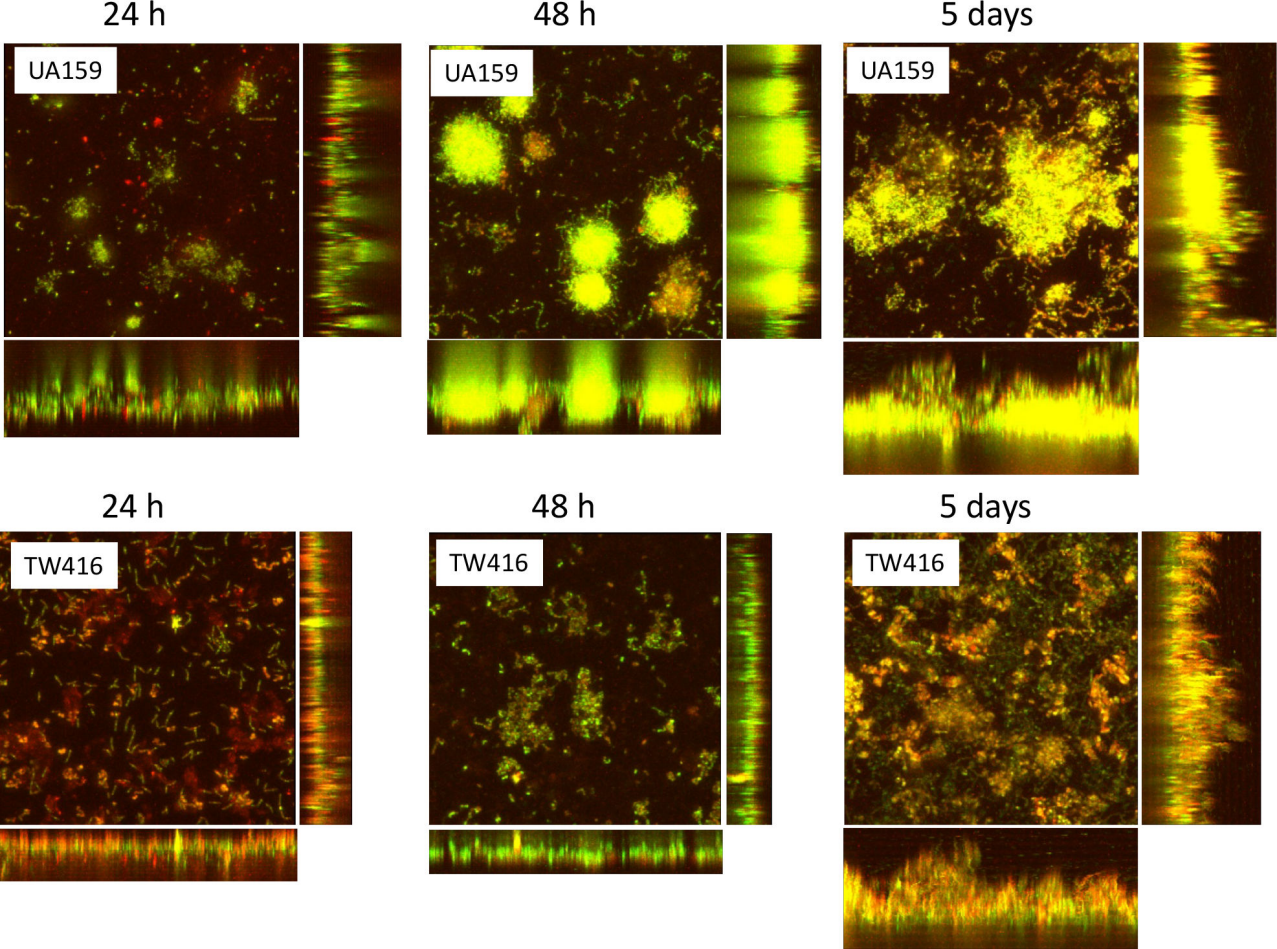
Confocal microscopy of mixed-species biofilms. The *S. mutans* wild-type UA159 and its allelic exchange *mecA* mutant, TW416 was grown in a mixed species consortium with *A. naeslundii*, *S. oralis,* and *L. casei. S. mutans* was tagged with a green fluorescence protein and at the end of 24 hours, 48 hours, and 5 days post-inoculation, the consortium biofilms were stained with a FM 1-43 dye, which confers all bacterial cells with red fluorescence. Images were representatives of compressed xyz, xz, and yz images (512 × 512).

**Fig 3:**
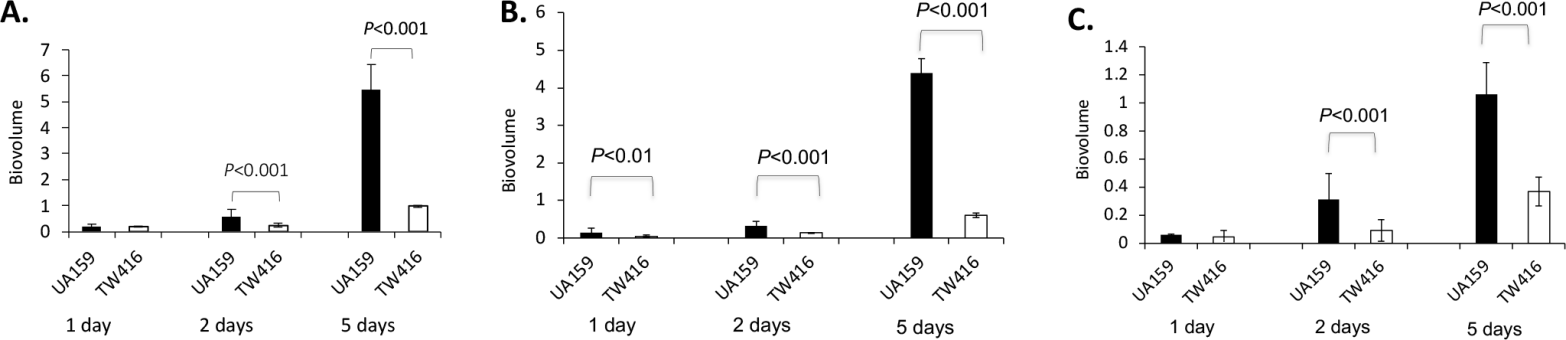
COMSTAT analysis of the mixed-species biofilms. Panels show (**A**) total biofilms in biovolume of the mixed-species consortium when grown with *S. mutans* UA159 and its *mecA* mutant, TW416; (**B**) biofilms of *S. mutans* UA159 and TW416 in the mixed-species consortium; and (**C**) biofilms of the other bacteria in the consortium with *S. mutans* UA159 and TW416. Data are expressed in µm^3^ µm^−2^ with *P* < 0.05 indicating statistical significance.

The impact of *S. mutans mecA* deficiency on multi-species biofilm development as a whole and the accumulation of the deficient mutant itself and other microorganisms in the mixed-species biofilm were evaluated. In a 96-well plate biofilm model (Fig. S2), there were no significant differences in the total biofilm biomass of the 24 h mixed-species biofilms between the cultures with the wild type, UA159, and those with the MecA-deficient mutant. Although total biofilm accumulation increased over 48 h and after 5 days for both the UA159- and the *mecA* mutant-associated cultures, the latter had significantly reduced the level of total biofilms at both time points, compared with the mixed-species biofilm with the wild-type UA159. Confocal microscopy was used to further observe the impact of MecA-deficiency on the biofilm architecture and the distribution of the different bacterial species in the polymicrobial community (Fig. 2). Interestingly, although co-cultivation with the *mecA* mutant led to a reduced accumulation of polymicrobial biofilms, the majority of the polymicrobial community still comprised *S. mutans*. This was evident by the widespread yellow color in the biofilms under the confocal microscope due to overlap between the green fluorescence-tagged *S. mutans* wild-type and *mecA* mutant cells and the red fluorescence of FM 1–43-stained cell membranes of all bacteria in the community. The wild-type cells formed large cell clusters while the *mecA* mutant formed loosely arranged smaller aggregates. COMSTAT analyses of the total mixed-species biofilm showed an increase in the biofilm biovolume (Fig. 3A) and biofilm thickness (Fig. S3A) over time, corroborating the 96-well plate biofilm results. Analysis of the microbial consortia in the mixed-species biofilm showed an increase of *S. mutans* wild-type and the *mecA* mutant biofilm biovolume (Fig. 3B) and thickness (Fig. S3B), indicating enhanced accumulation of the *S. mutans* cells over time. However, in comparison to UA159, the biovolume and thickness of the *mecA* mutant, TW416, were shown to be significantly reduced after 48 h and 5 days. The other members of the mixed-species community also accumulated and increased their biovolume (Fig. 3C) and biofilm thickness (Fig. S3C) over time, but notably, significantly more accumulation was observed in the mixed-species biofilms containing the *S. mutans* wild type.

### MecA deficiency in *S. mutans* reduces extracellular glucan production

**Fig 4:**
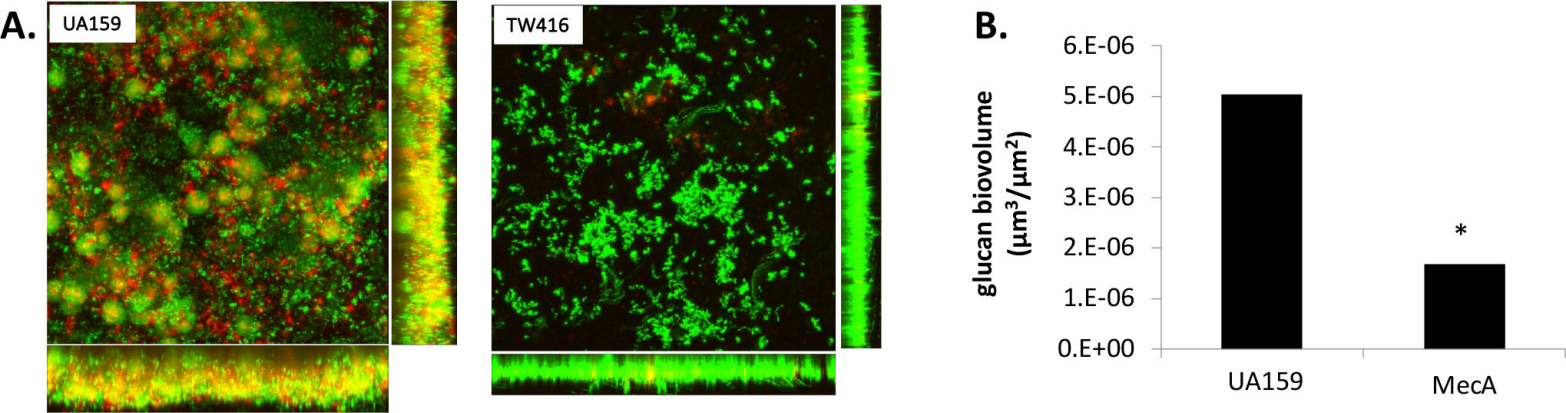
Glucan production of the MecA-deficient mutant. (**A**) Images of fluorescently labeled glucans (in red) produced by *S. mutans* wild type (UA159) and its *mecA* mutant (TW416) using confocal microscopy and (**B**) quantification of the glucan biovolume using COMSTAT. *, *P* < 0.05.

The effect of MecA*-*deficiency on *S. mutans* glucans production in 24 h biofilms grown on HA discs in the presence of 20 mM sucrose was analyzed using Alexa-Fluor 647 conjugated dextran (Fig. 4A). In comparison, UA159 biofilms had significantly more extracellular polysaccharides, a.k.a. glucans, compared with the *mecA* mutant biofilm. When further analyzed using COMSTAT, the *mecA* mutant biofilms had >3-fold decrease in the glucan level (*P* < 0.05, Fig. 4B).

### MecA deficiency affected *S. mutans* growth and biofilm formation differently from the Clp-deficient mutants

**Fig 5:**
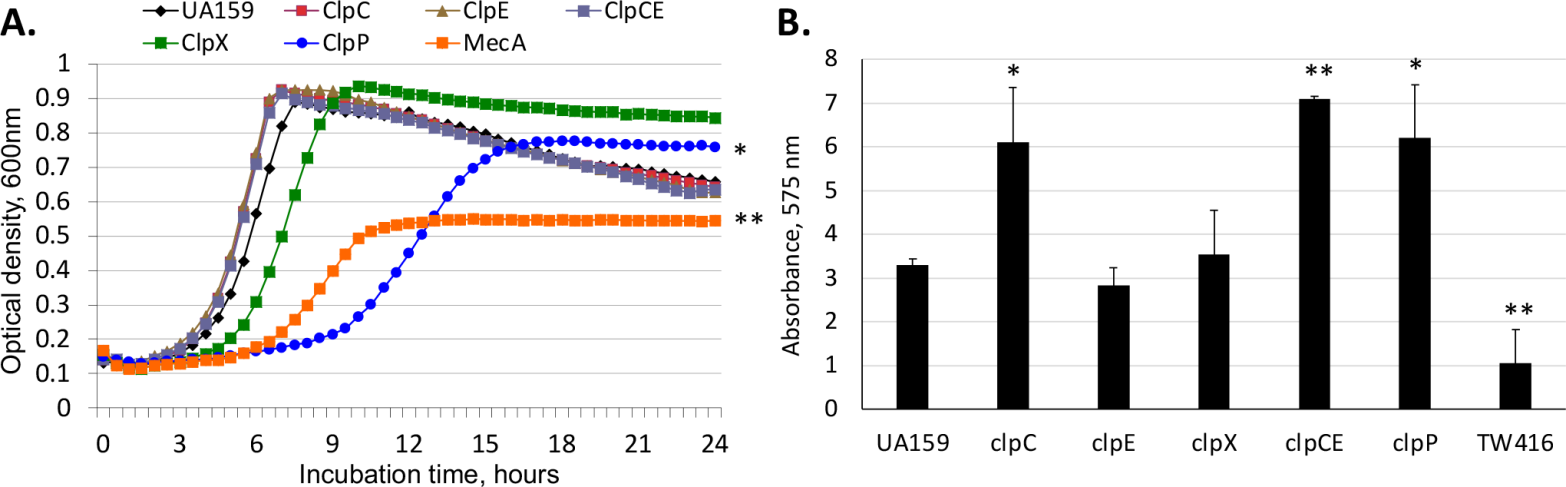
Growth and biofilm formation of the *S. mutans clp* mutants. (**A**) *S. mutans* wild-type UA159 and its *clpC, clpE, clpCE, clpX, clpP,* and *mecA* (TW416) mutants when grown in Bioscreen C in regular BHI broth. * and ** indicate *P* < 0.05 and 0.01, respectively, compared with UA159 in growth rate and optical density by Student’s *t*-test. (**B**) Twenty-four-hour biofilms of *S. mutans* UA159 and its *clpC, clpE, clpX, clpCE, clpP,* and *mecA* (TW416) mutants grown in BM-glucose plus sucrose. * and ** indicate *P* < 0.05 and 0.01, respectively, compared with UA159.

The growth phenotypes of the *mecA* mutant and the *clp* mutants was observed in the presence of BHI using Bioscreen C (Fig. 5A). Compared with the wild type, UA159, the *clpC*, *clpE,* and *clpCE* mutants all showed a slightly higher growth rate*,* whereas the *clpX* mutant had a slightly longer lag phase (around 4 h) compared with the wild type but reached a similar optical density by the end of the log phase. The *clpP* mutant had an extended lag phase (around 9 h) and reached an optical density slightly less than the wild type after 24 h. Different from the wild type and the *clp* mutants, the *mecA* mutant had an extended log phase (around 6 h), with a significantly lower growth rate. It reached a final optical density of around 0.55, about half of the value of the wild type as well as the *clp* mutants. The *mecA* complement strain restored the phenotype to levels similar to the wild type in growth rate and culture density. When grown on 96-well plates in BMGS, significantly more biofilms were measured with the *clpC-, clpCE-,* and *clpP*-deficient mutants, compared with the wild type, UA159, whereas the *clpE* and *clpX* mutants showed no major changes in biofilm formation (Fig. 5B). The allelic exchange *mecA* mutant, on the other hand, showed a significant decrease in biofilm formation, consistent with our previous findings (28).

When analyzed under transmission electron microscopy, the *clpP* mutant cells appeared to be slightly bigger in size than the parent strain, UA159, but there were no giant cells with multiple asymmetric septa (Fig. S4), as were commonly seen with the mutant with *mecA* deletion (28).

### Proteomics analysis revealed a unique set of proteins altered in the *mecA* mutant but not in the *clpP* mutant

When analyzed using proteomics, the *mecA* mutant was found to possess a proteome substantially different from the wild type, UA159, with more than 210 proteins upregulated by >1.5-fold (*P* < 0.05) and 125 proteins downregulated by >1.5-fold (*P* < 0.05) (Table S3a). Of the downregulated proteins, glycosyltransferase GtfBC&D and high-affinity adhesin P1, glucan-binding protein GbpC, surface-associated protein WapA and WapE, molecular chaperone GroES, and amyloid forming protein SMU.63C all are known to play a role in cellular biology and biofilm formation. The upregulated proteins include fructosyltransferase (Ftf), beta-exo-fructanase FruA, rhamnose-glucose polymer synthesis pathway RgpBDE, and F-ATPase subunits AtpABCDEFG, which are encoded in an operon, and cysteine desulfurase SufS of the SUF machinery (Table S3b).

When compared with the wild type, many proteins were also found to be differentially expressed in the ClpP-deficient mutant, including 134 downregulated and 142 upregulated by >1.5-fold (*P* < 0.05) (Table S3). Among the downregulated proteins are superoxide dismutase SOD, GtfD, trigger factor RopA, and members of the Tn*Smu1* island (Table S3c); the upregulated proteins include GtfB, ATPases ClpC, ClpE and ClpX, MecA, and molecular chaperones GroES and GroEL and their transcriptional repressors CtsR and HcrA (Table S3d).

**Fig 6:**
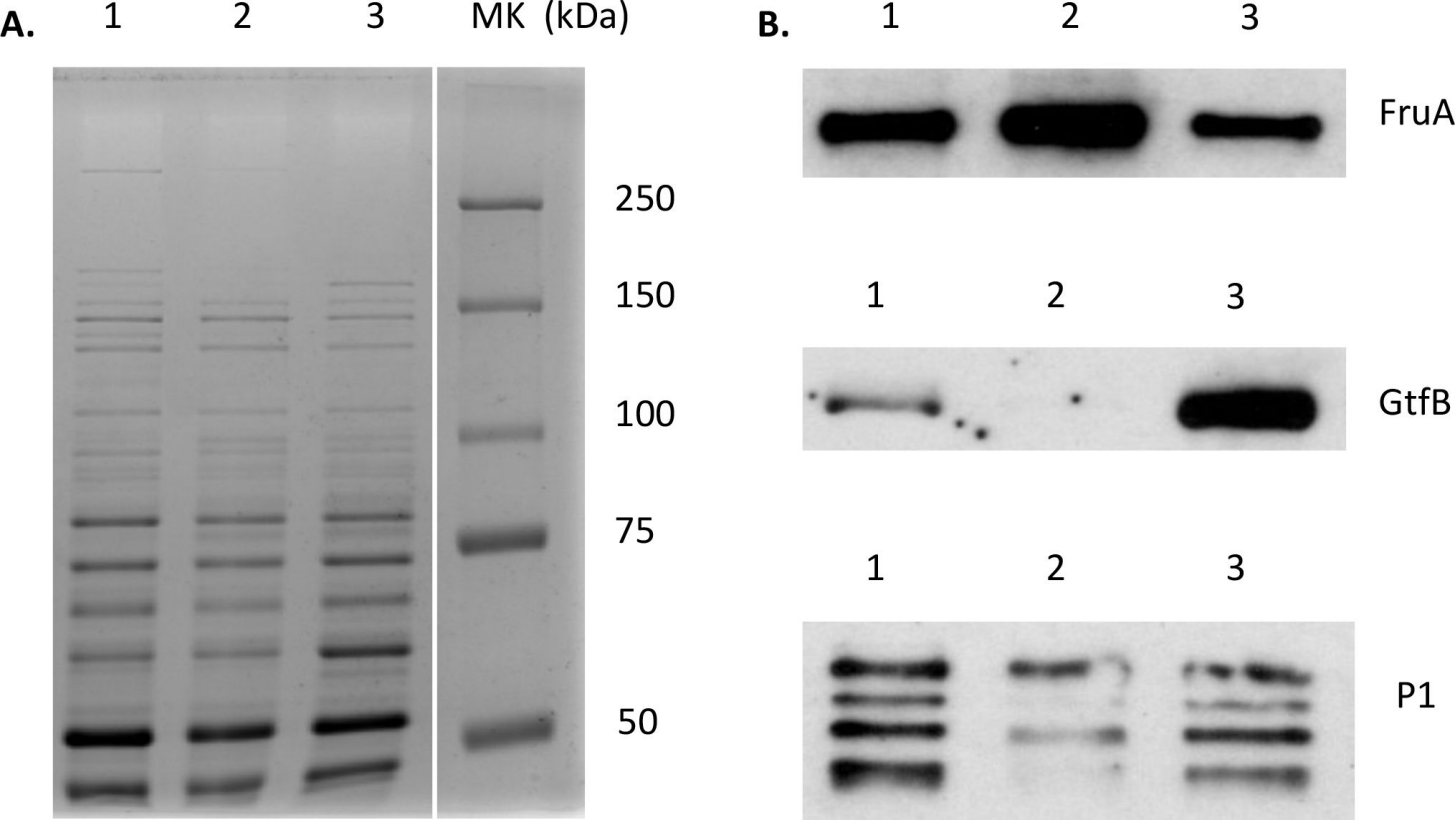
Western blot analyses. Whole cell lysates (10 µg total proteins) of *S. mutans* wild type, UA159 (lane 1), and its MecA-deficient mutant (TW416, lane 2) and ClpP-deficient mutant (lane 3) were separated using a 7.5% SDS-PAGE gel stained with Coomassie blue (A), blotted to a PVDF membrane, and then probed using polyclonal (FruA) or monoclonal (for GtfB and P1) antibodies (**B**).

As expected, when the two proteomic profiles were compared, many proteins were identified as consistently up- or down-regulated in both the *mecA* mutant and the *clpP* mutant including the downregulation of WapA, WapE, and GtfD. Many proteins, including ATPases ClpC, E, and X, molecular chaperones GroES and GroEL, and the repressor CtsR and HcrA, were found to be significantly altered in the *clpP* mutant but not in the *mecA*. Interestingly, there were also large groups of proteins that were altered in the *mecA* mutant but not in the *clpP* mutant or in a trend opposite to the *clpP* mutant (Table S4). Among them were FruA, GtfB, and P1. When further analyzed using western blotting, GtfB was shown to be significantly reduced in the *mecA* mutant (Fig. 6), which is also consistent with our previous study (28), whereas it was increased by >5-fold in the *clpP* mutant as shown by Kajfasz et al. (29). A major reduction of P1 was shown in the *mecA* mutant, which is also consistent with our previous study (28), but no major differences were observed between the *clpP* mutant and the wild type (Fig. S4). Consistently, as shown by western blotting, the *mecA* mutant displayed significant increases in FruA expression (Fig. 6), whereas the *clpP* mutant did not. These results further suggest that MecA is more than an adapter, and it also influences protein expression independently of the ClpP protease.

### Affinity pull-down and proteomics identified proteins interactive with MecA

**Fig 7:**
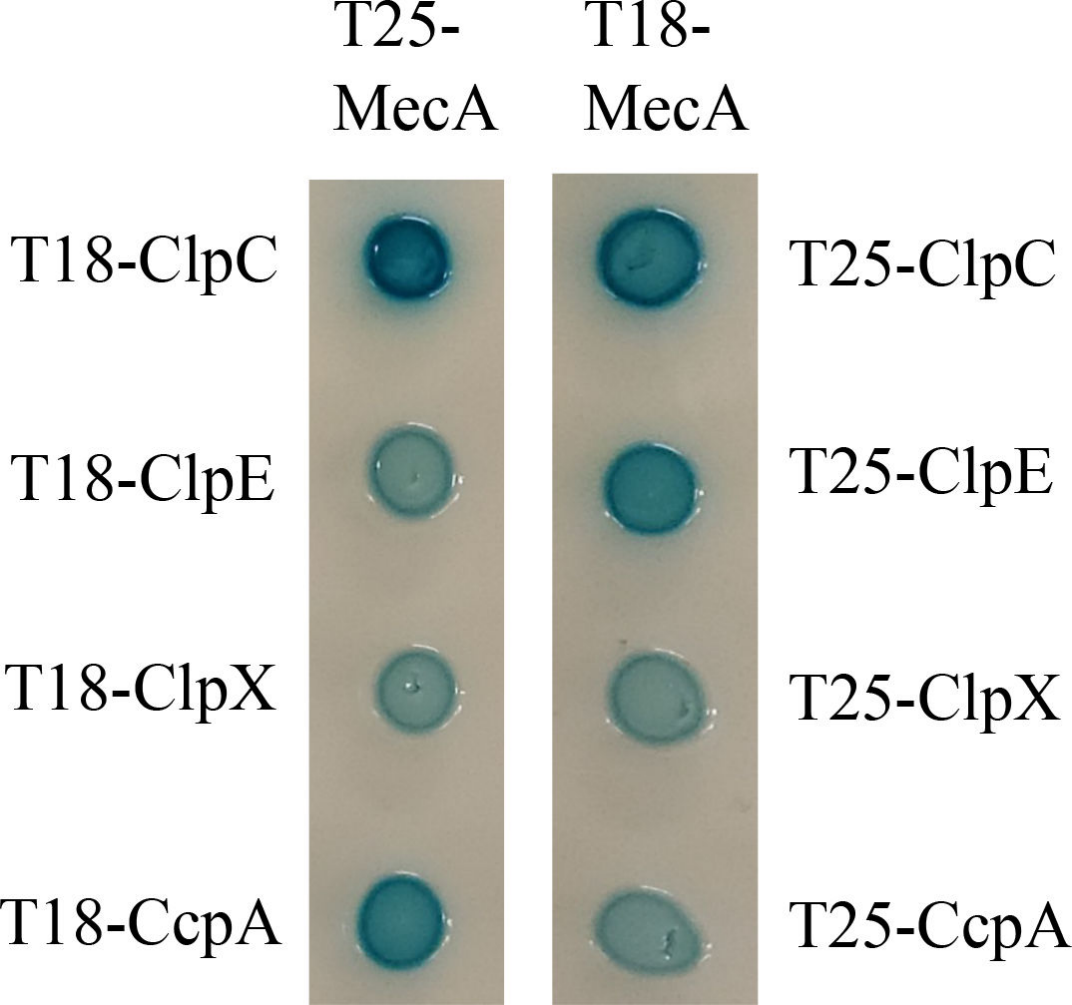
Bacterial two-hybrid analysis of the MecA interactome. Interactions between MecA and its putative partners were assessed using constructs cloned into plasmids pKT25 and pUT18C. Positive interactions were observed between MecA and ATPase ClpE and ClpX and transcription regulator CcpA, when fused to both the T18 and T25 fragment of the adenylate cyclase Cya, as indicated by formation of blue colonies. ATPase ClpC was serving as a reference.

Using affinity pull-down with rMecA as the bait, more than 30 proteins were identified in the rMecA complex by LC/MS, including ATPases ClpC, ClpE and ClpX, and CcpA, a global regulator of sugar metabolism and many other cellular processes (Table S5). To determine if ClpE, ClpX, and CcpA are part of the MecA interactome, the BACTH bacteria-two-hybrid system was used with *mecA* and its putative partner *clpE, clpX,* and *ccpA* fused with the catalytic domain T18 in pUT18 and domain T25 in pKT25, respectively, and with *clpC* serving as a reference. Following sequencing confirmation, the recombinant plasmids were co-transformed into the *E. coli* reporter strain, BTH101, and the co-transformants were checked on LB agar plates plus X-Gal and IPTG. The results showed that strong blue colonies were developed when the CcpA was fused to the N-terminal region of the T18 fragment (Fig. 7), whereas the blue colonies were relatively lighter when CcpA was fused to the T25 domain at its C-terminus. These results indicate complementation of Cya^+^ phenotype and protein-protein interactions between MecA and CcpA, and such interactions are likely at the N-terminal region of the regulatory protein (Fig. 7). Similarly, strong blue colonies were observed when the respective ClpE- and ClpX-fusion constructs were co-transformed with the MecA fusions, indicative of ClpX and ClpE as members of MecA interactome.

## DISCUSSION

MecA, a highly conserved protein in low GC Gram-positive bacteria, is known as an adapter protein of the regulated proteolytic machinery with important roles in protein homeostasis. In *S. mutans,* we recently showed that mutants with deletion of *mecA* led to major growth defects, especially when growing in the presence of stressors, defects in cell division, and alteration of cell morphology. Consistent with our previous study, which used allelic exchange mutagenesis, the results presented here showed that the frameshift mutant resulting from a single nucleotide deletion displayed the same growth and morphological characteristics as the allelic exchange mutant. This, along with the fact that carrying a copy of the wild-type *mecA* gene plus its cognate promoter *in trans* fully complements the *mecA* mutant (28), confirms that the phenotypes observed with the *mecA* mutants are direct results from *mecA* deletion/mutation and not because of any potential polar effects on the downstream *rgpG,* which encodes the first enzyme of biosynthesis pathway of the important rhamnose-containing glucose polymers (RGP) (28, 46). These results also provided further evidence that MecA in *S. mutans* plays critical roles in cellular biology and biofilms formation and that major differences exist between the *mecA* mutant and the *clpP* mutant.

*S. mutans* lives almost exclusively in the highly diverse and dynamic microbiota on the tooth surface. Many factors are well known to play a role in bacterial adherence and/or accumulation on a surface. As revealed by proteomics analysis and further confirmed by western blotting for some, downregulation of the Gtf enzymes, especially GtfB, and the high-affinity adhesin P1 are likely some of the major factors that underlie the reduced biofilm formation by the *mecA* mutant. Consistently, the *mecA* mutant also displayed a major reduction in its glucan production, as was shown by confocal microscopy with Alexa-Fluor dextran conjugate. Considering the role of the extracellular polysaccharides of *S. mutans* in the establishment and accumulation of mixed-species biofilms, the reduction of biofilm formation by the other bacteria when growing in communities with the *mecA* mutant can also be in part attributed to the reduced ability of the *mecA* mutant to produce glucans, compared to those with the wild type. Other downregulated proteins including glucan-binding protein GbpC, surface-associated protein WapA and WapE, molecular chaperones GroEL and GroES, amyloid- forming protein SMU.63C, the RGP biosynthesis enzyme RgpG, glutathione reductase GshR, and autoinducer LuxS are also known to play a role in *S. mutans* physiology and biofilm formation and, therefore, are likely part of the contributing factors to the defects of the *mecA* mutant in biofilm formation.

It is well established that an adapter of the Clp proteolytic machinery, MecA forms complexes with ATPase ClpC, which recognizes and unfolds target substrate proteins and translocates them to protease ClpP for degradation (18, 20). All streptococci (except *S. pneumoniae*) possess five Clp ATPases but have only one ClpP protease (47, 48). Unlike *B. subtilis* which possesses multiple adapters (49, 50)*,* MecA is the sole adapter protein that can be identified in *S. mutans* (47). Recent studies by Tian et al*.* have shown that adapter MecA interacts with ClpC in regulation of genetic competence development (21). Results of affinity pulldown assay and bacterial two hybrid analysis presented here further demonstrated that besides ClpC, MecA also interacts with ATPase ClpX and ClpE, two other ATPases with distinct tripeptidase sequences required for ClpP interactions and formation of Clp complexes. This is also consistent with a recent report by Gurung and Biswas (51), which reports that a ClpX motif was identified in MecA. Recent studies have also shown that ATPase ClpC, ClpE, and ClpX all play an important but slightly different roles in *S. mutans* physiology and regulation of virulence properties (13, 14, 29, 51, 52). Therefore, it is not surprising that the *clpC, clpX,* and *clpE* mutants do not share resemblance with the *mecA* mutant in growth characteristics and biofilm formation. However, the distinct phenotypes in growth, cell division, and biofilm formation observed between the *mecA* mutant and the *clpP* mutant as well as the distinct proteomic profile of the *mecA* mutant also suggest that MecA can regulate *S. mutans* physiology and virulence properties including biofilm formation through alternate pathways independent of the ClpP protease (28). In support of this notion, similar phenomena have also recently been reported in *B. subtilis* (53). In *B. subtilis,* MecA was shown *in vitro* to interact with phosphorylated transcriptional regulator Spo0A, which regulates sporulation and biofilm formation, and such interactions also require ClpC. However, unlike the adaptor MecA, such interaction is not accompanied by degradation of the regulator. Instead, the MecA-ClpC complex was thought to directly bind to the target promoters, influencing transcription of target genes (53), although currently there is no further information available concerning the ClpP-independent regulation of gene expression in *S. mutans* or any other bacteria.

To uncover the scope of the MecA-mediated, ClpP-independent regulation in *S. mutans,* affinity pulldown and investigative proteomics were used. Affinity pulldown identified more than 31 proteins, which include many that were identified in the proteomes of the *mecA* and *clpP* mutants, and among the interactome is also CcpA. Carbon catabolite repressor CcpA in *S. mutans* is a major transcription regulator that governs the regulation of multiple metabolic pathways including sugar metabolism such as the utilization of the β-(2→1) linked fructose polymers (a.k.a. fructans) via fructanase FruA (54, 55). As a transcriptional regulator, CcpA possesses an alpha-helix turn-helix DNA-binding motif in its N-terminus. The B2H results show that strong interaction with MecA was observed with the N-terminal T18 fusion but only weakly with the C-terminal T25 fusion suggesting that MecA likely interacts with the N-terminal region of CcpA, influencing regulation of its target genes. Consistently, the elevated expression of FruA in the *mecA* mutant, but not the *clpP* mutant, as shown by proteomics analysis and western blotting, also suggests that the MecA-mediated regulation of FruA expression is ClpP-independent; and it is likely at the transcriptional level via direct interactions with transcriptional repressor CcpA. Otherwise, if the MecA-CcpA interaction is ClpP dependent, *clpP* deletion would be expected to lead to accumulation of CcpA, which in turn results in reduced *fruA* expression. However, further investigation via *in vitro* transcription with and without inclusion of CcpA and MecA, along with *in vitro* assay involving ClpC/P-mediated degradation will provide more definite information on the underlying mechanisms of how MecA regulates FruA expression in *S. mutans*.

It is known that the regulated Clp proteolysis is critical for the maintenance of cellular homeostasis, which includes unfolding and degradation of enzymes and regulatory proteins (13, 14). Consistently, the levels of many groups of proteins were found to be altered in response to ClpP-deficiency, including ATPases ClpE, ClpX, and ClpC and adapter MecA (all upregulated). Like *B. subtilis,* adapter MecA facilitates ClpC oligomerization and thus targeting and presenting substrates to protease ClpP barrel for degradation, where MecA is hydrolyzed along with the substrate target. Therefore, accumulation of MecA in the *clpP* deletion mutant is as expected. On the other hand, the elevation of ClpE, ClpX, and ClpC is likely a compensatory effect in response to ClpP deficiency. Other elevated proteins in the *clpP* mutant also included those for stress tolerance responses (DnaK, DnaJ, GroEL, GroES, CtsR, and HrcA, all upregulated) and metabolic pathways (such as the glycogen biosynthesis proteins glgC&D, SMU.209/210, pyruvate dehydrogenase PdhAB, pyruvate formate-lyase Pfl, β-glucosidase BglA, and fructosyltransferase Ftf). It is worth noting that the proteomic profile of the *clpP* mutant is also very similar to the results of DNA microarray analysis of a *clpP* mutant by Chong et al. (48). As expected, the proteomic profile of the *mecA* mutant showed a lot of similarities to the *clpP* mutant with many proteins consistently up- or down-regulated in both the *mecA* and the *clpP* mutant. These proteins are likely substrates of the adapter MecA and the Clp proteolytic machinery.

It is noteworthy that during the preparation of this manuscript, the Merritt group at Oregon Health and Science University, Portland, OR used untargeted co-immunoprecipitation assays along with spectrometry and found that MecA has an interactome of >100 proteins, including many as part of highly conserved metabolic pathways. Using a newly developed prokaryotic split luciferase complementation assay, many of the interactions between MecA and its partners were further verified *in vivo* (56). The abundance of selected proteins in a *mecA* mutant and a *clpP/mecA* double mutant was further assessed using western blotting, and the results showed that more than half of the MecA partners tested were independent of the Clp proteolysis. Consistent with the results of affinity pulldown and proteomics analysis that we present here, the results of the Merritt group also suggest that MecA likely regulates much, if not most, of its diverse interactome through a Clp-independent pathway (56). Like *B. subtilis* (53) and as suggested by its interactions with transcriptional repressor CcpA, MecA in *S. mutans* likely regulates protein expression via interactions with transcriptional regulators. Further studies using *in vitro* transcription assay along with *in vitro* degradation assay and other related functional assays will be needed to pinpoint how exactly MecA interacts with CcpA and other proteins in the regulation of *S. mutans* physiology and virulence properties.

## SUPPLEMENTAL MATERIAL

Supplemental material msphere.00308-24-s0001.pdf Supplemental tables and figures.

ASM does not own the copyrights to Supplemental Material that may be linked to, or accessed through, an article. The authors have granted ASM a non-exclusive, world-wide license to publish the Supplemental Material files. Please contact the corresponding author directly for reuse.
