# Supplementary material for: MecA in Streptococcus mutans is a multi-functional protein
Source: mSphere. 2024 Nov 12;9(12):e00308-24. doi: 10.1128/msphere.00308-24 (PMC11656736; doi:10.1128/msphere.00308-24)
Supplement: Supplemental material — Supplemental tables and figures. [file msphere.00308-24-s0001.pdf]

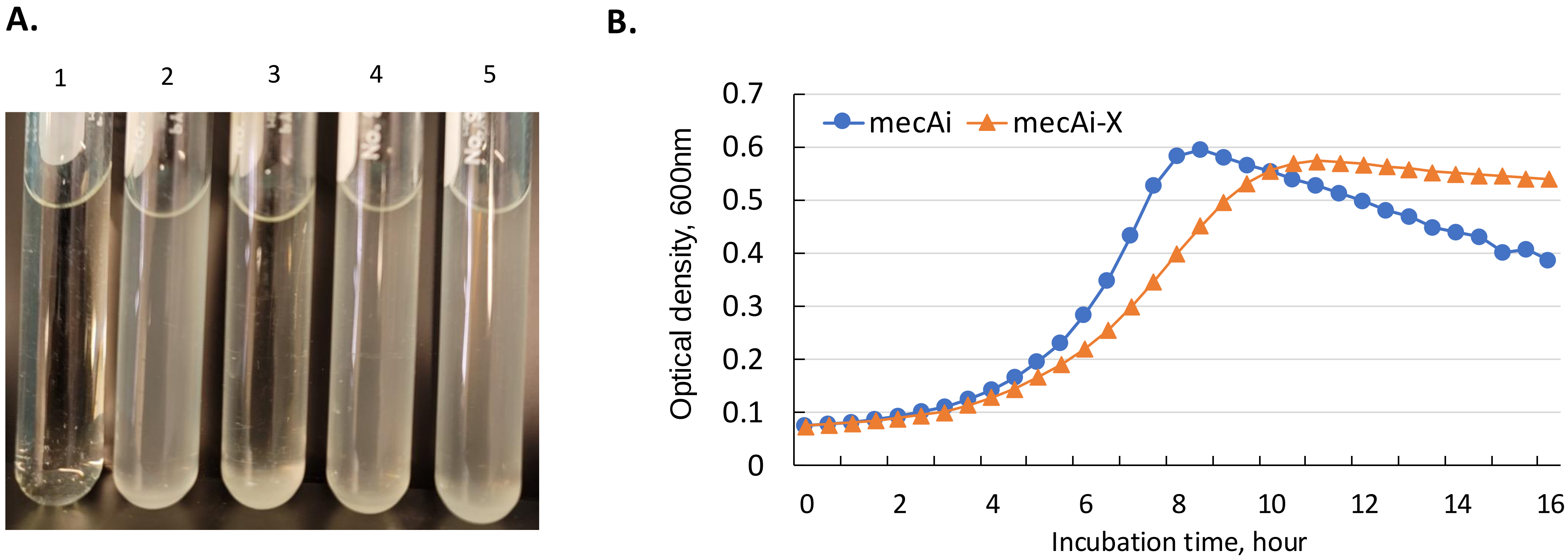

Fig. S1. (A). CRISPRi *mecA* (2,3) and *clpP* (4,5) mutants growing in defined medium FMC with (3,5) and without (2,4) inclusion of 0.2% xylose (w/v) with blank medium (1) as negative control. (B). Bioscreen C analysis of CRISPRi *mecA* mutant (mecAi) growing in FMC broth with and without xylose (X).

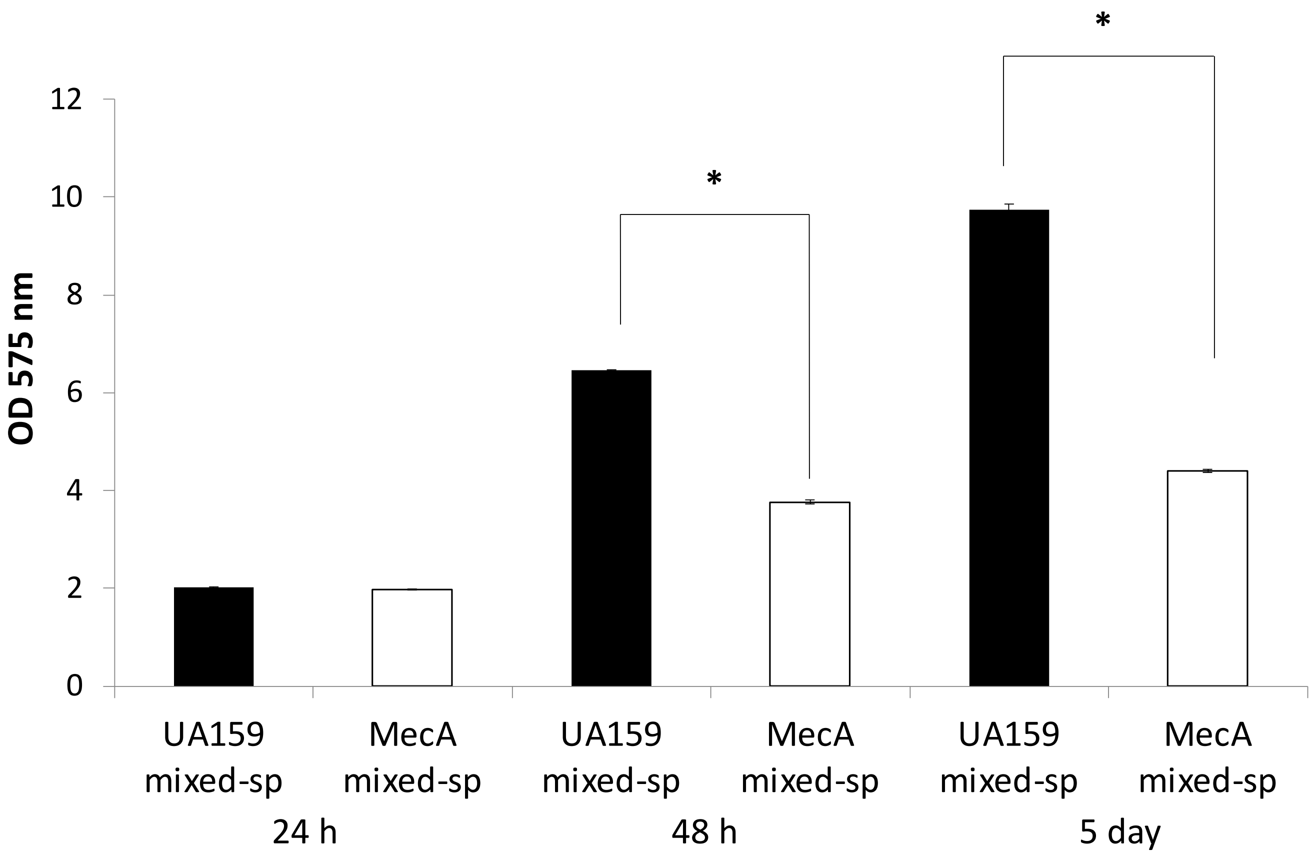

Fig S2: Mixed-species biofilm formation with *S. mutans* wild-type and its *mecA* mutant when grown in BM medium supplemented with 18 mM glucose and 2 mM sucrose in 96-well plates. Results showed that the *mecA* mutant-containing mixed-species consortium had significantly less biofilms, compared to wild type UA159 containing mixed-biofilms at 48 h and 5 days post inoculation. *, $P<0.05$, via student's *t* test.

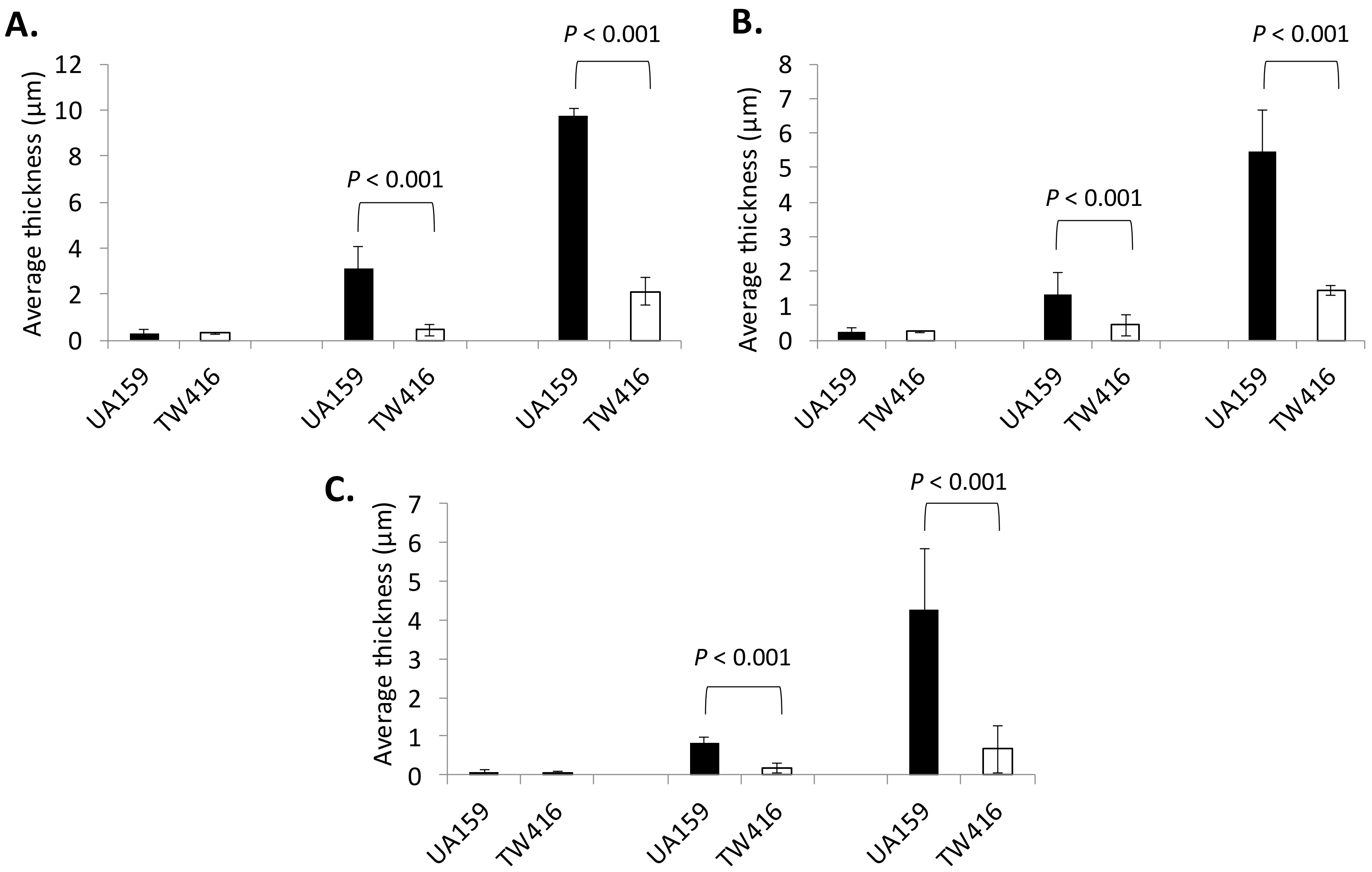

Fig. S3. COMSTAT analysis of biofilms of *S. mutans* UA159 and its *mecA* mutant, TW416 when grown in multiple species consortia. The thickness of (A) total biofilm, (B) *S. mutans* UA159 and TW416 biofilms, and (C) biofilms of the other bacteria in the consortium with UA159 and TW416 was evaluated. $P < 0.05$ indicate statistical significance.

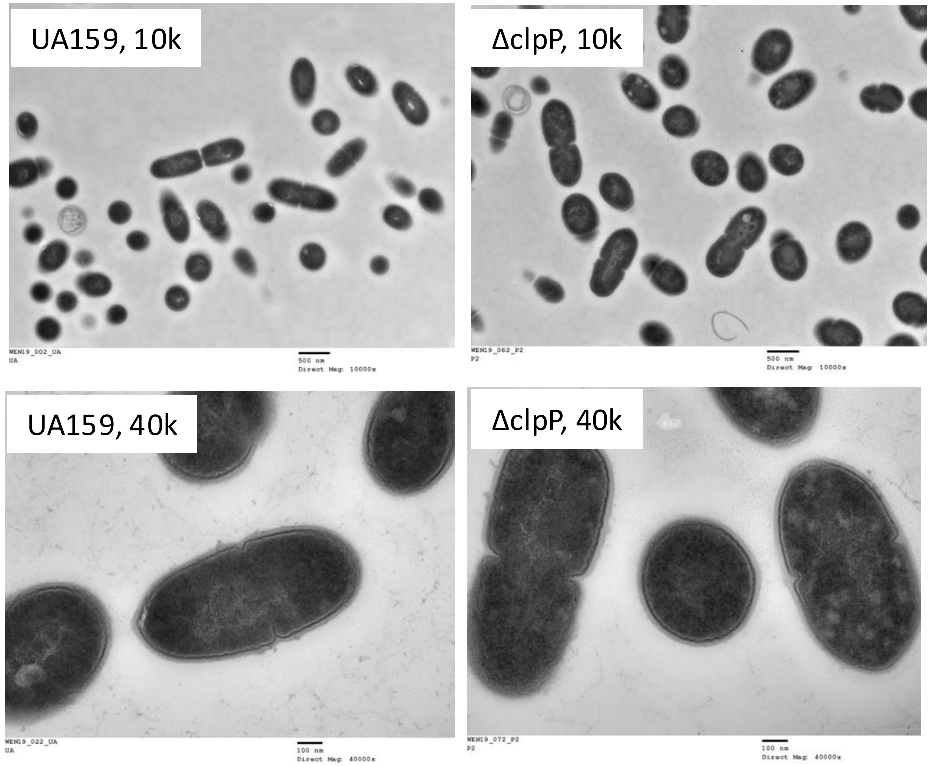

Fig. S4. TEM analysis of *S. mutans* wild-type UA159 and its *clpP* mutant (ΔclpP) when grown in regular BHI broth.

Table S1. Primers used in this study

Names	Oligonucleotide sequence (5'-3')	Application
mecAi-F	tcttttcctgGTTTTAGAGCTAGAAATAGC	CRISPRi sgRNA for *mecA*
mecAi-R	cggaagaattACATTTATTGTACAACACG	
clpPi-F	aaacgagccgGTTTTAGAGCTAGAAATAGC	CRISPRi sgRNA for *clpP*
clpPi-R	gttcaataacACATTTATTGTACAACACG	
mecAe-F	aagactggatccatggaaatgaaacaaatcagcgaaacaac	*mecA* expression
mecAe-R	actaaaggtcgacaaacaagtgaagtc	
clpX-F	atctagaaaatagaaccaatgatgtcac	two hybrid analysis of ClpX
clpX-R	acggtaccatctccataggaagctgtc	
ccpA-F	atctagacacagatgacacaatcac	two hybrid analysis of CcpA
ccpa-R	atggtacctcatatttagttgttcctctttc	
ccpA-R2	taggtaccttagttgttcctctttctctgataccat	
mecA-F	gctctagagatggaaatgaaacaaatcagc	two hybrid analysis of MecA
mecA-R	tgggtacccggtatcatctagcttatccaatc	
mecA-R2	taggtaccccaatcatttgtaattcttgcagag	
clpC-F	gctctagagatgaccgattactcattaaa	two hybrid analysis of ClpC
clpC-R	ggggtacccgacactgaagtagggtaaag	
clpE-F	ttaaaggatccgactattcatctatatgctaatg	two hybrid analysis of ClpE
clpE-R	taggtacctgatttctataatctgaag	

Note: Sequences underlined are restriction sites engineered for cloning.

Table S2. Doubling times and optical densities of the mutants

Strain name	Doubling time (hours)	P-value	Maximum OD at 600 nm	P-value
S. mutans UA159	1.79(±0.11)	0.0303	0.97 (±0.04)	0.0099
S. mutans TW416fs	2.88(±0.21)		0.59 (±0.05)	
S. mutans UA159 - pH 6	4.05(±0.03)	0.0017	0.90 (±0.01)	0.0017
S. mutans TW416fs - pH 6	5.57(±0.14)		0.60 (±0.01)	
S. mutans UA159 - MV	5.11(±0.28)	0.0001	0.63 (±0.02)	0.0312
S. mutans TW416fs - MV	19.15(±0.56)		0.15 (±0.003)	
S. mutans clpC	1.53(±0.06)	0.1238*	0.92(±0.02)	0.1951*
S. mutans clpE	1.59(±0.05)	0.1804*	0.92(±0.11)	0.5941*
S. mutans clpCE	1.57(±0.05)	0.1623*	0.91(±0.10)	0.3225*
S. mutans clpX	1.88(±0.07)	0.4311*	0.93(±0.04)	0.7625*
S. mutans clpP	4.99(±0.10)	0.0015*	0.77(±0.03)	0.0298*
S. mutans TW416	2.84(±0.14)	0.0134*	0.54(±0.03)	0.0196*

Note: The doubling times and optical densities were measured under the conditions as indicated and expressed as the averages (±standard deviation) of at least three separate sets of experiments. MV, Methyl viologen. *, P value as compared to *S. mutans* UA159 under the same conditions analyzed.

Table S3a. Down-regulated proteins identified in the *mecA* mutant*

Gene ID	Gene Symbol	Description /putative function	Ratio (M/U)	P-Value
SMU_09	SMU_09	hypothetical protein SMU_09	0.591	0.01406
SMU_1004	gtfB	Glucosyltransferase-I	0.576	0.00000
SMU_1005	gtfC	Glucosyltransferase-SI	0.646	0.00004
SMU_1091	wapE	cell wall protein, WapE	0.364	0.00001
SMU_1132	pepN	aminopeptidase	0.556	0.00031
SMU_1175	SMU_1175	Sodium/amino acid (Alanine) symporter	0.522	0.04059
SMU_119	adh	Alcohol dehydrogenase	0.611	0.00040
SMU_1214	pyrC	dihydroorotase	0.545	0.00059
SMU_1247	eno	Enolase	0.539	0.00000
SMU_1268	hisB	imidazoleglycerol-phosphate dehydratase	0.315	0.00805
SMU_1271	hisG	ATP phosphoribosyltransferase	0.606	0.02882
SMU_1282	SMU_1282	transcriptional regulator	0.601	0.00018
SMU_1287	SMU_1287	transcriptional regulator	0.578	0.00021
SMU_1297	SMU_1297	hypothetical protein SMU_1297	0.478	0.00001
SMU_1298	rl31	50S ribosomal protein L31	0.586	0.00051
SMU_1339	bacD	bacitracin synthetase	0.407	0.00003
SMU_1340	bacA2	Surfactin synthetase	0.413	0.00000
SMU_1341c	SMU_1341c	Gramicidin S synthetase	0.389	0.00000
SMU_1342	bacA1	bacitracin synthetase 1; BacA	0.372	0.00000
SMU_1343c	SMU_1343c	Polyketide synthase	0.352	0.00000
SMU_1344c	SMU_1344c	malonyl CoA-ACP transacylase	0.345	0.00000
SMU_1345c	SMU_1345c	peptide synthetase	0.329	0.00000
SMU_1346	bacT	thioesterase	0.280	0.00007
SMU_1361c	SMU_1361c	TetR family transcriptional regulator	0.225	0.00001
SMU_1365c	SMU_1365c	permease	0.170	0.00000
SMU_1366c		ABC transporter ATP-binding protein	0.182	0.00000
SMU_1377c	SMU_1377c	hypothetical protein SMU_1377c	0.479	0.00000
SMU_1383	leuB	3-isopropylmalate dehydrogenase	0.539	0.00000
SMU_1384	leuA	2-isopropylmalate synthase	0.522	0.00000

SMU_1396	gbpC	glucan-binding protein gbpC	0.325	0.00000
SMU_1443c	SMU_1443c	tributyrin esterase	0.630	0.00227
SMU_1470c	SMU_1470c	hypothetical protein SMU_1470c	0.418	0.00102
SMU_1492	lacF	PTS system lactose-specific transporter subunit IIA	0.401	0.00000
SMU_1496	lacA	Galactose-6-phosphate isomerase subunit lacA	0.423	0.03776
SMU_1510	syfB	phenylalanyl-tRNA synthetase subunit beta	0.541	0.00000
SMU_1512	syfA	phenylalanyl-tRNA synthetase subunit alpha	0.648	0.04892
SMU_1542c	SMU_1542c	lipid kinase	0.554	0.00724
SMU_155	pnpA	polynucleotide phosphorylase	0.588	0.00008
SMU_158	cysS	cysteinyl-tRNA synthetase	0.544	0.01126
SMU_1586	syt1	threonyl-tRNA synthetase	0.553	0.00001
SMU_1626	rl1	50S ribosomal protein L1	0.623	0.04272
SMU_1627	rl11	50S ribosomal protein L11	0.426	0.00000
SMU_1632	pfs	5'-methylthioadenosine/S-adenosylhomocysteine nucleosidase	0.625	0.00344
SMU_1736	accC	acetyl-CoA carboxylase biotin carboxylase subunit	0.523	0.00821
SMU_1739	fabF	3-oxoacyl-ACP synthase	0.452	0.00000
SMU_1742c	SMU_1742c	trans-2-enoyl-ACP reductase	0.636	0.00014
SMU_1743	acp	Acyl carrier protein	0.392	0.01695
SMU_1799	nadD	nicotinic acid mononucleotide adenylyltransferase	0.523	0.00000
SMU_1819	gatB	aspartyl/glutamyl-tRNA amidotransferase subunit B	0.651	0.00010
SMU_1821c	SMU_1821c	aspartyl/glutamyl-tRNA amidotransferase subunit C	0.649	0.00048
SMU_1823	pncA	Pyrazinamidase/nicotinamidase	0.368	0.00009
SMU_1831	aspG	L-asparaginase	0.506	0.00001
SMU_1834	alr	Alanine racemase	0.547	0.01842
SMU_1836	aroG	phospho-2-dehydro-3-deoxyheptonate aldolase	0.449	0.00000
SMU_1837	aroH	phospho-2-dehydro-3-deoxyheptonate aldolase	0.459	0.00000
SMU_184	sloC	ABC transporter metal binding lipoprotein	0.534	0.00000
SMU_1840	scrK	fructokinase	0.650	0.03722
SMU_1849	comEB	deoxycytidylate deaminase	0.576	0.01155
SMU_1859	ssb	single-stranded DNA-binding protein	0.499	0.04007
SMU_1860	rs6	30S ribosomal protein S6	0.515	0.00025
SMU_1877	ptnA	PTS system mannose-specific transporter subunit IIAB	0.664	0.00019

SMU_1924	gcrR	response regulator GcrR for glucan-binding protein C	0.611	0.01684
SMU_1943	syl	leucyl-tRNA synthetase	0.460	0.00000
SMU_1955	groES	co-chaperonin GroES	0.485	0.00022
SMU_1973	pepA	glutamyl-aminopeptidase; endo-1,4-beta-glucanase	0.445	0.00095
SMU_1992	tyrS	tRNA ligase; Tyrosyl-tRNA synthetase	0.440	0.00000
SMU_2000	rl17	50S ribosomal protein L17	0.576	0.00000
SMU_2003a		50S ribosomal protein L36	0.267	0.00000
SMU_2008	rl30	50S ribosomal protein L30	0.527	0.00427
SMU_2009	rs5	30S ribosomal protein S5	0.628	0.03212
SMU_2011	rl6	50S ribosomal protein L6	0.539	0.00021
SMU_2016	rl24	50S ribosomal protein L24	0.433	0.00010
SMU_2019	rl29	50S ribosomal protein L29	0.526	0.00455
SMU_2022	rl22	50S ribosomal protein L22	0.587	0.03333
SMU_2031	eftS	Elongation factor Ts	0.647	0.00000
SMU_2037	treA	trehalose-6-phosphate hydrolase TreA	0.398	0.00000
SMU_2088	ruvA	holliday junction DNA helicase RuvA	0.488	0.03146
SMU_2139c	SMU_2139c	50S ribosomal protein L9	0.437	0.00000
SMU_2147c	SMU_2147c	hypothetical protein SMU_2147c	0.122	0.00000
SMU_2166	SMU_2166	50S ribosomal protein L23	0.664	0.00413
SMU_218	SMU_218	transcriptional regulator	0.469	0.00022
SMU_236c	SMU_236c	transcriptional regulator	0.544	0.04992
SMU_246	rgpG	glycosyl transferase N-acetylglucosaminyltransferase RgpG	0.136	0.00000
SMU_317	SMU_317	tetrahydrodipicolinate succinylase	0.553	0.00000
SMU_325	SMU_325	Deoxyuridine 5'-triphosphate nucleotidohydrolase	0.451	0.00000
SMU_338	SMU_338	RNA-binding protein	0.514	0.00001
SMU_352	SMU_352	ribulose-phosphate 3-epimerase	0.568	0.00000
SMU_360	gapC	Extracellular glyceraldehyde-3-phosphate dehydrogenase	0.508	0.00001
SMU_393	SMU_393	hypothetical protein SMU_393	0.626	0.00007
SMU_395	pepX	X-prolyl-dipeptidyl aminopeptidase	0.470	0.01200
SMU_466	pepC	cysteine aminopeptidase	0.502	0.00000
SMU_474	luxS	S-ribosylhomocysteinase	0.405	0.00014
SMU_494	SMU_494	fructose-6-phosphate aldolase	0.568	0.00975

SMU_495	gldA	Glycerol dehydrogenase	0.499	0.00013
SMU_51	purK	phosphoribosylaminoimidazole carboxylase ATPase subunit	0.630	0.02050
SMU_542	glk	Glucose kinase	0.577	0.00000
SMU_558	SMU_558	isoleucyl-tRNA synthetase	0.509	0.00452
SMU_573	SMU_573	hypothetical protein SMU_573	0.593	0.00050
SMU_595	pyrD	dihydroorotate dehydrogenase 1A	0.541	0.03504
SMU_610	spaP	cell surface antigen SpaP	0.527	0.00000
SMU_63c	SMU_63c	hypothetical protein SMU_63c	0.593	0.00093
SMU_672	idh	isocitrate dehydrogenase	0.665	0.00780
SMU_675	SMU_675	PTS system transporter protein EI	0.646	0.00000
SMU_684	SMU_684	hypothetical protein SMU_684	0.606	0.01303
SMU_689	SMU_689	hypothetical protein SMU_689	0.346	0.00000
SMU_697	SMU_697	translation initiation factor IF-3	0.595	0.02457
SMU_718c	SMU_718c	hypothetical protein SMU_718c	0.586	0.00000
SMU_73	SMU_73	hypothetical protein SMU_73	0.403	0.00012
SMU_730	SMU_730	hypothetical protein SMU_730	0.604	0.00193
SMU_759	SMU_759	protease	0.458	0.02247
SMU_81	grpE	Heat shock protein GrpE	0.568	0.00000
SMU_838	gshR	glutathione reductase	0.510	0.00626
SMU_849	SMU_849	50S ribosomal protein L27	0.507	0.00020
SMU_862	SMU_862	permease	0.339	0.00000
SMU_865	SMU_865	30S ribosomal protein S16	0.439	0.00025
SMU_871	pfkB	fructose-1-phosphate kinase	0.619	0.03718
SMU_91	ropA	trigger factor	0.605	0.00000
SMU_910	gtfD	glucosyltransferase-S	0.464	0.00204
SMU_913	SMU_913	glutamate dehydrogenase	0.389	0.00000
SMU_957	SMU_957	50S ribosomal protein L10	0.444	0.00000
SMU_960	rplL	50S ribosomal protein L7/L12	0.514	0.00001
SMU_987	wapA	Cell wall-associated protein WapA	0.472	0.01712
SMU_99	fbaA	fructose-bisphosphate aldolase	0.620	0.00000
SMU_990	dapA	dihydrodipicolinate synthase	0.627	0.00179

* Data are presented in ratios of the abundance of the *mecA* mutant (M) over the abundance of the wild-type (U). Only proteins with a P value <0.05 and a reduction of >1.5-fold are presented.

Table S3b. Up-regulated proteins identified in the *mecA* mutant*

Gene ID	Gene Symbol	Description /putative function	Ratio (M/U)	P-Value
SMU_08	trcF	transcription-repair coupling factor	1.529	0.00008
SMU_1001	smf	DNA processing Smf protein	2.914	0.01388
SMU_1009	SMU_1009	histidine kinase	1.948	0.00002
SMU_1017	oadB	oxaloacetate decarboxylase, sodium ion pump subunit	2.567	0.01475
SMU_1037c	SMU_1037c	histidine kinase	2.128	0.00062
SMU_1041	SMU_1041	ABC transporter ATP-binding protein	2.308	0.00004
SMU_1043c	SMU_1043c	Phosphotransacetylase	1.812	0.00000
SMU_1045c	SMU_1045c	inorganic polyphosphate/ATP-NAD kinase	1.564	0.00000
SMU_1052	SMU_1052	hypothetical protein SMU_1052	2.098	0.00000
SMU_1054	SMU_1054	glutamine amidotransferase	1.590	0.01040
SMU_1067c	SMU_1067c	ABC transporter permease	4.036	0.00000
SMU_1068c	SMU_1068c	ABC transporter ATP-binding protein	4.157	0.00000
SMU_1069c	SMU_1069c	hypothetical protein SMU_1069c	3.752	0.00319
SMU_1070c	SMU_1070c	hypothetical protein SMU_1070c	3.452	0.00354
SMU_1078c	SMU_1078c	ABC transporter ATP-binding protein	1.940	0.00000
SMU_1083c	SMU_1083c	hypothetical protein SMU_1083c	1.830	0.00132
SMU_1084	hemK	N5-glutamine S-adenosyl-L-methionine-dependent methyltransferase	2.362	0.02277
SMU_1088	apbE	thiamine biosynthesis lipoprotein	1.542	0.03197
SMU_1089	SMU_1089	hypothetical protein SMU_1089	1.607	0.00000
SMU_1090	SMU_1090	hypothetical protein SMU_1090	1.870	0.00014
SMU_1096	opuBa	choline transporter ABC transporter ATP-binding protein	1.562	0.00001
SMU_1118c	SMU_1118c	ABC sugar transporter, permease	2.435	0.02279
SMU_1119c	SMU_1119c	Sugar ABC transporter permease	3.798	0.00154
SMU_1120	SMU_1120	sugar ABC transporter ATP-binding protein	2.472	0.00000
SMU_114	SMU_114	PTS system fructose-specific transporter subunit IIBC	5.604	0.00080
SMU_115	SMU_115	PTS system fructose-specific transporter subunit IIA	10.106	0.00033
SMU_1169c	SMU_1169c	thioredoxin	3.802	0.00033
SMU_1178c	SMU_1178c	Amino acid ABC transporter ATP-binding protein	2.002	0.02875
SMU_1180	phnA	alkylphosphonate uptake protein	3.355	0.00076

SMU_1182	mtlD	Mannitol-1-phosphate 5-dehydrogenase	1.789	0.04564
SMU_1183	mtlA2	PTS system mannitol-specific transporter subunit IIA	5.073	0.00000
SMU_1188	lepB	signal peptidase	1.567	0.00000
SMU_1194	SMU_1194	ABC transporter ATP-binding protein	1.640	0.00000
SMU_12	SMU_12	hypothetical protein SMU_12	3.551	0.01988
SMU_1204	parC	DNA topoisomerase IV subunit A	1.723	0.00234
SMU_1210	parE	DNA topoisomerase IV subunit B	1.501	0.00023
SMU_1245c	SMU_1245c	hypothetical protein SMU_1245c	2.557	0.00138
SMU_1256c	SMU_1256c	hypothetical protein SMU_1256c	3.733	0.00000
SMU_1269	serB	phosphoserine phosphatase	2.399	0.00643
SMU_1276c	SMU_1276c	septation ring formation regulator EzrA	1.575	0.00014
SMU_129	adhC	branched-chain alpha-keto acid dehydrogenase E2 subunit	1.815	0.00441
SMU_1302	adcA	surface adhesin	2.287	0.00000
SMU_1306c	SMU_1306c	glmZ(sRNA)-inactivating NTPase	1.524	0.01563
SMU_1307c	SMU_1307c	hypothetical protein SMU_1307c	1.911	0.00079
SMU_1324	ftsX	cell-division protein FtsX	1.755	0.00067
SMU_1325	ftsE	ABC transporter ATP-binding protein	1.792	0.00000
SMU_1389	pckA	hypothetical protein SMU_1389	1.557	0.00000
SMU_1391c	SMU_1391c	hypothetical protein SMU_1391c	1.619	0.00140
SMU_1412c	SMU_1412c	ABC transporter subunit and ATP-binding protein	1.749	0.00061
SMU_1421	pdhC	branched-chain alpha-keto acid dehydrogenase E2 subunit	11.413	0.00001
SMU_1422	pdhB	Pyruvate dehydrogenase E1 component subunit beta	9.189	0.00205
SMU_1423	pdhA	Pyruvate dehydrogenase, TPP-dependent E1 component alpha-subunit	7.368	0.00130
SMU_1442c	SMU_1442c	hypothetical protein SMU_1442c	1.976	0.00015
SMU_1467	apt	Adenine phosphoribosyltransferase	1.663	0.00000
SMU_1473c	SMU_1473c	Oxidoreductase	2.135	0.00001
SMU_148	adhE	bifunctional acetaldehyde-CoA/alcohol dehydrogenase	2.465	0.00000
SMU_1480	SMU_1480	hypothetical protein SMU_1480	1.832	0.01200
SMU_1487	SMU_1487	hypothetical protein SMU_1487	1.698	0.00694
SMU_1513	smc	Chromosome segregation ATPase	1.603	0.03113
SMU_1527	atpA	ATP synthase F0F1 subunit epsilon	1.997	0.00014
SMU_1528	atpB	ATP synthase F0F1 subunit beta	2.082	0.00000

SMU_1529	atpC	ATP synthase F0F1 subunit gamma	2.240	0.00144
SMU_1530	atpD	ATP synthase F0F1 subunit alpha	2.174	0.00000
SMU_1531	atpE	ATP synthase F0F1 subunit delta	2.390	0.00000
SMU_1532	atpF	ATP synthase F0F1 subunit B	2.240	0.00000
SMU_1533	atpG	ATP synthase F0F1 subunit A	2.031	0.01222
SMU_1537	glgD	glycogen biosynthesis protein glgD	2.306	0.00001
SMU_1538	glgC	Glucose-1-phosphate adenylyltransferase; glucose pyrophosphorylase	2.216	0.00189
SMU_1539	glgB	glycogen branching protein	3.520	0.02819
SMU_1563	pacL	Cation-transporting P-type ATPase PacL	2.412	0.00000
SMU_1568	malX	maltose ABC transporter substrate-binding protein	2.935	0.00000
SMU_1570	malG	maltose ABC transporter permease	3.262	0.00021
SMU_1571	SMU_1571	MsmK-like ABC transporter ATP-binding protein	2.963	0.00064
SMU_1576c	SMU_1576c	hypothetical protein SMU_1576c	2.279	0.00041
SMU_1577c	SMU_1577c	hypothetical protein SMU_1577c	2.186	0.01119
SMU_1603	lguL	lactoylglutathione lyase	2.358	0.00098
SMU_1613c	SMU_1613c	dephospho-CoA kinase	2.630	0.00019
SMU_1616c	SMU_1616c	hypothetical protein SMU_1616c	4.071	0.00094
SMU_1621c	SMU_1621c	hypothetical protein SMU_1621c	4.043	0.00009
SMU_1622	pmsR	methionine sulfoxide reductase A	2.607	0.02828
SMU_1641c	SMU_1641c	hypothetical protein SMU_1641c	2.849	0.00000
SMU_1644c	SMU_1644c	hypothetical protein SMU_1644c	1.661	0.00002
SMU_1665	livF	branched chain amino acid ABC transporter ATP-binding protein	1.647	0.00026
SMU_1666	livG	branched chain amino acid ABC transporter ATP-binding protein	1.641	0.00226
SMU_1678	SMU_1678	acyl-CoA thioesterase	1.775	0.00000
SMU_1688	dltD	extramembranal protein, DltD protein	1.992	0.00094
SMU_1712c	SMU_1712c	segregation and condensation protein B	1.860	0.00344
SMU_1716c	SMU_1716c	hypothetical protein SMU_1716c	2.200	0.00000
SMU_1725	SMU_1725	acylphosphatase	2.686	0.01915
SMU_1771c	SMU_1771c	hypothetical protein SMU_1771c	3.176	0.00011
SMU_1777	nrdI	flavoprotein NrdI	2.790	0.00000
SMU_1788c	SMU_1788c	bacterocin transport accessory protein, Bta	3.729	0.00000
SMU_1789c	SMU_1789c	hypothetical protein SMU_1789c	1.584	0.00055

SMU_1841	scrA	PTS system sucrose-specific transporter subunit IIABC	2.069	0.00044
SMU_1855	SMU_1855	hypothetical protein SMU_1855	1.645	0.00002
SMU_1869	trxA	thioredoxin	2.534	0.00000
SMU_1878	ptnC	PTS system mannose-specific transporter subunit IIC	1.673	0.00015
SMU_1902c	SMU_1902c	hypothetical protein SMU_1902c	3.717	0.00025
SMU_1923c	SMU_1923c	transcriptional regulator NrdR	1.552	0.00000
SMU_1935c	SMU_1935c	hypothetical protein SMU_1935c	1.571	0.00005
SMU_1939c	SMU_1939c	ABC transporter ATP-binding protein	1.702	0.04661
SMU_1957	SMU_1957	PTS system mannose-specific transporter subunit IID	2.946	0.00063
SMU_1958c	SMU_1958c	PTS system mannose-specific transporter subunit IIC	3.613	0.00000
SMU_1960c	SMU_1960c	PTS system mannose-specific transporter subunit IIB	3.714	0.00000
SMU_1961c	SMU_1961c	PTS system sugar-specific transporter subunit IIA	1.603	0.00000
SMU_1971c	SMU_1971c	thioredoxin	3.149	0.03997
SMU_1975c	SMU_1975c	hypothetical protein SMU_1975c	1.817	0.03582
SMU_20	mreC	Cell shape-determining protein MreC	4.297	0.00000
SMU_2005	adk	adenylate kinase	2.652	0.00000
SMU_2021	rs3	30S ribosomal protein S3	1.507	0.00262
SMU_2027	SMU_2027	transcriptional regulator	2.648	0.00000
SMU_2028	sacB	Beta-D-fructosyltransferase, Ftf	3.618	0.00000
SMU_2047	ptsG	PTS system glucose-specific transporter subunit IIABC	1.734	0.00000
SMU_2057c	SMU_2057c	Cadmium-transporting ATPase	3.295	0.04937
SMU_205c	SMU_205c	hypothetical protein SMU_205c	3.981	0.01102
SMU_2074	nrdD	anaerobic ribonucleoside triphosphate reductase	1.525	0.00000
SMU_2079c	SMU_2079c	hypothetical protein SMU_2079c	1.558	0.00424
SMU_209c	SMU_209c	hypothetical protein SMU_209c	3.426	0.00000
SMU_210c	SMU_210c	hypothetical protein SMU_210c	2.935	0.00008
SMU_2121c	SMU_2121c	hypothetical protein SMU_2121c	2.823	0.00000
SMU_2129c	SMU_2129c	hypothetical protein SMU_2129c	2.110	0.00000
SMU_2137c	SMU_2137c	hypothetical protein SMU_2137c	1.657	0.00981
SMU_227c	SMU_227c	hypothetical protein SMU_227c	1.693	0.00004
SMU_250	nifU	nitrogen fixation-like protein, NifU	1.837	0.00007
SMU_255	oppA	Oligopeptide ABC transporter substrate-binding protein OppA	1.602	0.00000

SMU_270	sgaT	PTS system ascorbate-specific transporter subunit IIC	1.941	0.00000
SMU_271	ptxB	PTS system transporter subunit IIB	2.941	0.00000
SMU_298	SMU_298	hypothetical protein SMU_298	2.589	0.00106
SMU_308	SMU_308	sorbitol-6-phosphate 2-dehydrogenase	3.733	0.01540
SMU_312	SMU_312	PTS system sorbitol phosphotransferase transporter subunit IIBC	4.349	0.00630
SMU_334	SMU_334	Argininosuccinate synthase	1.711	0.00000
SMU_35	purN	Phosphoribosylglycinamide formyltransferase	5.325	0.00024
SMU_354	SMU_354	hypothetical protein SMU_354	1.612	0.04306
SMU_384	SMU_384	hypothetical protein SMU_384	1.741	0.02164
SMU_386	SMU_386	ribosomal-protein-alanine acetyltransferase	4.199	0.00000
SMU_396	glpF	glycerol uptake facilitator protein	1.598	0.00528
SMU_399	SMU_399	hypothetical protein SMU_399	1.666	0.00003
SMU_402	pfl	pyruvate formate-lyase	3.098	0.00000
SMU_412c	SMU_412c	cell-cycle regulation protein	2.303	0.00000
SMU_428	SMU_428	hypothetical protein SMU_428	2.110	0.01194
SMU_438c	SMU_438c	(R)-2-hydroxyglutaryl-CoA dehydratase activator	2.353	0.02850
SMU_440	SMU_440	hypothetical protein SMU_440	2.942	0.00000
SMU_455	pbp2x	Penicillin-binding protein 2X	1.830	0.00000
SMU_458	SMU_458	ATP-dependent RNA helicase	1.536	0.00100
SMU_459	SMU_459	ABC transporter amino acid binding protein	2.080	0.00291
SMU_473	SMU_473	hypothetical protein SMU_473	1.565	0.02019
SMU_503c	SMU_503c	hypothetical protein SMU_503c	7.628	0.00001
SMU_508	SMU_508	hypothetical protein SMU_508	1.707	0.00810
SMU_510c	SMU_510c	hypothetical protein SMU_510c	4.774	0.00000
SMU_518	SMU_518	hypothetical protein SMU_518	3.108	0.00000
SMU_527	SMU_527	hypothetical protein SMU_527	1.631	0.00261
SMU_528c	SMU_528c	hypothetical protein SMU_528c	4.225	0.00000
SMU_549	murG	undecaprenyldiphospho-muramoylpentapeptide beta-N-acetylglucosaminyltransferase	1.662	0.01790
SMU_557	divIVA	cell division protein DivIVA	1.747	0.00125
SMU_561c	SMU_561c	hydrolase (MutT family)	3.583	0.00004
SMU_567	SMU_567	Glutamine ABC transporter permease	2.145	0.00378
SMU_568	SMU_568	Amino acid ABC transporter ATP-binding protein	1.800	0.00002

SMU_577	lytS	histidine kinase LytS	1.769	0.01220
SMU_585	recN	DNA repair protein RecN	1.644	0.01112
SMU_597	pbp2b	penicillin-binding protein 2B	2.777	0.00000
SMU_609	SMU_609	40K cell wall protein	11.617	0.00000
SMU_633	SMU_633	thioesterase	2.112	0.00000
SMU_636	SMU_636	N-acetylglucosamine-6-phosphate isomerase	2.768	0.00000
SMU_648	prtM	foldase PrsA	1.576	0.00146
SMU_65	SMU_65	Protein tyrosine-phosphatase	1.818	0.00000
SMU_668c	SMU_668c	ribonucleotide-diphosphate reductase subunit alpha	1.764	0.00001
SMU_669c	SMU_669c	glutaredoxin	3.524	0.00000
SMU_674	ptsH	phosphocarrier protein HPr	2.809	0.00000
SMU_678	SMU_678	Oxidoreductase	1.828	0.00348
SMU_685	SMU_685	hypothetical protein SMU_685	2.058	0.00093
SMU_695	SMU_695	hypothetical protein SMU_695	1.848	0.01771
SMU_696	SMU_696	cytidylate kinase	1.701	0.00330
SMU_723	SMU_723	Cadmium-transporting ATPase	1.509	0.00004
SMU_746c	SMU_746c	hypothetical protein SMU_746c	1.914	0.00136
SMU_769	SMU_769	hypothetical protein SMU_769	2.910	0.00006
SMU_775c	SMU_775c	hypothetical protein SMU_775c	1.917	0.00033
SMU_78	fruA	Exo-beta-D-fructosidase	1.372	0.00000
SMU_785	aroK	shikimate kinase	3.205	0.00000
SMU_790	SMU_790	hypothetical protein SMU_790	3.101	0.00422
SMU_794	SMU_794	hypothetical protein SMU_794	3.213	0.00009
SMU_796	SMU_796	hypothetical protein SMU_796	2.087	0.00000
SMU_804	SMU_804	hypothetical protein SMU_804	2.154	0.00521
SMU_815	SMU_815	amino acid ABC transporter substrate-binding protein	1.572	0.00039
SMU_819	mscL	large conductance mechanosensitive channel	1.865	0.00013
SMU_824	rlmD	dTDP-4-keto-L-rhamnose reductase	1.614	0.00000
SMU_826	rgpB	Rhamnosyltransferase	1.630	0.02510
SMU_828	rgpD	polysaccharide ABC transporter ATP-binding protein	1.578	0.00000
SMU_829	rgpE	glycosyltransferase	1.607	0.03964
SMU_832	SMU_832	hypothetical protein SMU_832	1.779	0.02478

SMU_833	SMU_833	glycosyltransferase	1.613	0.00235
SMU_837	SMU_837	reductase	2.977	0.00000
SMU_872	SMU_872	PTS system fructose-specific transporter subunit IIABC	1.552	0.01811
SMU_878	msmE	Multiple sugar-binding ABC transporter, sugar-binding protein MsmE	4.121	0.00072
SMU_882	msmK	multiple sugar-binding ABC transporter ATP-binding protein, MsmK	4.337	0.00000
SMU_883	dexB	Dextran glucosidase DexB	3.519	0.00782
SMU_891	hsdM	type I restriction-modification system DNA methylase	1.770	0.00858
SMU_892	hsdS	type I restriction-modification system, specificity determinant	1.966	0.03697
SMU_893	SMU_893	Anticodon nuclease	2.300	0.03533
SMU_905	SMU_905	ABC transporter ATP-binding protein	2.464	0.00000
SMU_923	SMU_923	ABC transporter ATP-binding protein	2.096	0.00837
SMU_924	tpx	lipid hydroperoxide peroxidase	3.126	0.00000
SMU_932	SMU_932	hypothetical protein SMU_932	3.464	0.01650
SMU_936	SMU_936	Amino acid ABC transporter ATP-binding protein	4.225	0.00000
SMU_950	SMU_950	GTP-binding protein YsxC	1.559	0.00000
SMU_972	murB	UDP-N-acetylenolpyruvoylglucosamine reductase	1.705	0.00000
SMU_984	SMU_984	hypothetical protein SMU_984	6.845	0.00003
SMU_985	bglA	beta-glucosidase	3.452	0.00000
SMU_991	SMU_991	ribonucleotide reductase	2.093	0.00261
SMU_998	SMU_998	ABC transporter periplasmic ferrichrome-binding protein	1.700	0.00011
SUM_96	rpoE	putative DNA-directed RNA polymerase, delta subunit	4.058	0.00000

* Data are presented in ratios of the abundance of the *mecA* mutant (M) over the abundance of the wild-type (U).

Only proteins with a P value <0.05 and a ratio >1.5 are presented.

Table S3c. Down-regulated proteins identified in the *clpP* mutant*

Gene ID	Gene Symbol	Description /putative function	Ratio (C/U)	P-Value
SMU_1083c	SMU_1083c	hypothetical protein SMU_1083c	0.563	0.00473
SMU_1091	wapE	cell wall protein, WapE	0.320	0.00000
SMU_1175	SMU_1175	Sodium/amino acid (Alanine) symporter	0.386	0.00233
SMU_1201c	SMU_1201c	hypothetical protein SMU_1201c	0.644	0.00000
SMU_1269	serB	phosphoserine phosphatase	0.510	0.03124
SMU_1271	hisG	ATP phosphoribosyltransferase	0.564	0.01210
SMU_1278c	SMU_1278c	hypothetical protein SMU_1278c	0.476	0.00000
SMU_1282	SMU_1282	transcriptional regulator	0.581	0.00007
SMU_1291c	SMU_1291c	hypothetical protein SMU_1291c	0.588	0.01938
SMU_1297	SMU_1297	hypothetical protein SMU_1297	0.659	0.00734
SMU_1298	rl31	50S ribosomal protein L31	0.566	0.00027
SMU_1315c	SMU_1315c	ATP-binding protein	0.617	0.02261
SMU_1339	bacD	bacitracin synthetase	0.411	0.00004
SMU_134	SMU_134	TetR/AcrR family transcriptional regulator	0.630	0.03712
SMU_1340	bacA2	Surfactin synthetase	0.125	0.00000
SMU_1341c	SMU_1341c	Gramicidin S synthetase	0.115	0.00000
SMU_1342	bacA1	bacitracin synthetase 1; BacA	0.126	0.00000
SMU_1343c	SMU_1343c	Polyketide synthase	0.122	0.00000
SMU_1344c	SMU_1344c	malonyl CoA-ACP transacylase	0.099	0.00000
SMU_1345c	SMU_1345c	peptide synthetase	0.105	0.00000
SMU_1346	bacT	thioesterase	0.082	0.00000
SMU_1361c	SMU_1361c	TetR family transcriptional regulator	0.315	0.00025
SMU_1365c	SMU_1365c	permease	0.084	0.00000
SMU_1366c		ABC transporter ATP-binding protein	0.150	0.00000
SMU_136c	SMU_136c	transcriptional regulator	0.112	0.00010
SMU_1377c	SMU_1377c	hypothetical protein SMU_1377c	0.463	0.00000
SMU_1417c	SMU_1417c	oleoyl-acyl carrier protein thioesterase	0.664	0.04334
SMU_1447c	SMU_1447c	hypothetical protein SMU_1447c	0.633	0.00151
SMU_1470c	SMU_1470c	hypothetical protein SMU_1470c	0.538	0.01657

SMU_1487	SMU_1487	hypothetical protein SMU_1487	0.442	0.00006
SMU_1490	lacG	6-phospho-beta-galactosidase	0.317	0.04245
SMU_1491	lacE	PTS system lactose-specific transporter subunit IIBC	0.427	0.00054
SMU_1492	lacF	PTS system lactose-specific transporter subunit IIA	0.233	0.00000
SMU_1493	lacD	tagatose 1,6-diphosphate aldolase	0.406	0.00132
SMU_1494	lacC	Tagatose-6-phosphate kinase	0.392	0.03064
SMU_1496	lacA	Galactose-6-phosphate isomerase subunit lacA	0.379	0.01726
SMU_1510	syfB	phenylalanyl-tRNA synthetase subunit beta	0.554	0.00000
SMU_1512	syfA	phenylalanyl-tRNA synthetase subunit alpha	0.641	0.04246
SMU_1520	SMU_1520	ABC transporter glutamine binding protein	0.479	0.00097
SMU_154	SMU_154	30S ribosomal protein S15	0.662	0.00000
SMU_1572	murZ	UDP-N-acetylglucosamine 1-carboxyvinyltransferase	0.584	0.00000
SMU_1586	syt1	threonyl-tRNA synthetase	0.534	0.00001
SMU_1595	cah	Carbonic anhydrase	0.308	0.00001
SMU_1624	rrf1	ribosome recycling factor	0.559	0.00367
SMU_1625	pyrH	uridylate kinase	0.583	0.00012
SMU_1641c	SMU_1641c	hypothetical protein SMU_1641c	0.548	0.00000
SMU_1664c	SMU_1664c	acetoin utilization protein, acetoin dehydrogenase	0.624	0.02210
SMU_1665	livF	branched chain amino acid ABC transporter ATP-binding	0.648	0.00151
SMU_1666	livG	branched chain amino acid ABC transporter ATP-binding	0.604	0.00216
SMU_1667	livM	Branched chain amino acid ABC transporter permease	0.490	0.00048
SMU_1668	livH	Branched chain amino acid ABC transporter permease	0.620	0.00000
SMU_1669	livK	Branched chain amino acid ABC transporter	0.481	0.00033
SMU_1673	upp	uracil phosphoribosyltransferase	0.636	0.00033
SMU_169	SMU_169	50S ribosomal protein L13	0.610	0.02267
SMU_1741	fabD	malonyl CoA-ACP transacylase	0.565	0.00001
SMU_1743	acp	Acyl carrier protein	0.420	0.03228
SMU_1777	nrdI	flavoprotein NrdI	0.665	0.00011
SMU_1788c	SMU_1788c	bacterocin transport accessory protein, Bta	0.402	0.00001
SMU_184	sloC	ABC transporter metal binding lipoprotein	0.526	0.00000
SMU_1858	rs18	30S ribosomal protein S18	0.659	0.00019
SMU_1860	rs6	30S ribosomal protein S6	0.603	0.00254

SMU_1877	ptnA	PTS system mannose-specific transporter subunit IIAB	0.441	0.00000
SMU_1879	SMU_1879	PTS system mannose-specific transporter subunit IID	0.503	0.00000
SMU_1924	gcrR	response regulator GcrR for glucan-binding protein C	0.620	0.02635
SMU_1931	gidB	16S rRNA methyltransferase GidB	0.603	0.00009
SMU_1941	atmB	Membrane lipoprotein	0.619	0.00007
SMU_1943	syl	leucyl-tRNA synthetase	0.659	0.00001
SMU_1960c	SMU_1960c	PTS system mannose-specific transporter subunit IIB	0.641	0.00008
SMU_1961c	SMU_1961c	PTS system sugar-specific transporter subunit IIA	0.512	0.00000
SMU_1963c	SMU_1963c	sugar-binding periplasmic protein	0.457	0.00042
SMU_1970c	SMU_1970c	phenylalanyl-tRNA synthetase subunit beta	0.653	0.03610
SMU_1973	pepA	glutamyl-aminopeptidase; endo-1,4-beta-glucanase	0.509	0.00614
SMU_1974	proC	Pyrroline-5-carboxylate reductase	0.613	0.00080
SMU_1979c	SMU_1979c	hypothetical protein SMU_1979c	0.600	0.00552
SMU_1992	tyrS	Tyrosine--tRNA ligase	0.441	0.00000
SMU_2003a		50S ribosomal protein L36	0.405	0.00000
SMU_2011	rl6	50S ribosomal protein L6	0.649	0.00871
SMU_2031	eftS	Elongation factor Ts	0.599	0.00000
SMU_2037	treA	trehalose-6-phosphate hydrolase TreA	0.445	0.00000
SMU_2079c	SMU_2079c	hypothetical protein SMU_2079c	0.647	0.00683
SMU_2104a	SMU_2104a	50S ribosomal protein L32	0.658	0.00240
SMU_2147c	SMU_2147c	hypothetical protein SMU_2147c	0.175	0.00000
SMU_2157	guaB	inosine 5'-monophosphate dehydrogenase	0.587	0.00000
SMU_218	SMU_218	transcriptional regulator	0.444	0.00009
SMU_231	ilvB	acetolactate synthase catalytic subunit	0.645	0.00000
SMU_232	ilvH	Acetolactate synthase 3 regulatory subunit	0.575	0.00000
SMU_233	ilvC	ketol-acid reductoisomerase	0.490	0.00000
SMU_241c	SMU_241c	Amino acid ABC transporter ATP-binding protein	0.404	0.00000
SMU_242c	SMU_242c	amino acid ABC transporter permease	0.390	0.00000
SMU_243	SMU_243	hypothetical protein SMU_243	0.586	0.00000
SMU_255	oppA	Oligopeptide ABC transporter substrate-binding protein OppA	0.639	0.00000
SMU_257	oppC	transmembrane permease OppC	0.658	0.00046
SMU_271	ptxB	PTS system transporter subunit IIB	0.649	0.00180

SMU_287	SMU_287	ComB, accessory factor for ComA	0.580	0.01144
SMU_291	tkt	Transketolase	0.665	0.00002
SMU_333	SMU_333	hypothetical protein SMU_333	0.493	0.00000
SMU_335	SMU_335	argininosuccinate lyase	0.518	0.00579
SMU_342	SMU_342	hypothetical protein SMU_342	0.584	0.00836
SMU_358	SMU_358	30S ribosomal protein S7	0.622	0.00040
SMU_364	glnA	glutamate--ammonia ligase	0.508	0.00000
SMU_393	SMU_393	hypothetical protein SMU_393	0.501	0.00000
SMU_396	glpF	glycerol uptake facilitator protein	0.550	0.00039
SMU_401c	SMU_401c	hypothetical protein SMU_401c	0.317	0.00011
SMU_408	SMU_408	permease	0.476	0.00000
SMU_420	SMU_420	hypothetical protein SMU_420	0.568	0.00004
SMU_450	proA	Gamma-glutamyl phosphate reductase	0.650	0.02609
SMU_501	SMU_501	hypothetical protein SMU_501	0.460	0.00000
SMU_502	SMU_502	hypothetical protein SMU_502	0.374	0.00000
SMU_595	pyrD	dihydroorotate dehydrogenase 1A	0.358	0.00031
SMU_629	sod	manganese-type superoxide dismutase, Fe/Mn-SOD	0.574	0.01876
SMU_66	SMU_66	hypothetical protein SMU_66	0.454	0.00956
SMU_689	SMU_689	hypothetical protein SMU_689	0.426	0.00000
SMU_697	SMU_697	translation initiation factor IF-3	0.615	0.03381
SMU_698	SMU_698	50S ribosomal protein L35	0.322	0.00044
SMU_721	SMU_721	hypothetical protein SMU_721	0.479	0.03857
SMU_768c	SMU_768c	hypothetical protein SMU_768c	0.522	0.03009
SMU_773c	SMU_773c	lysyl-tRNA synthetase	0.591	0.00016
SMU_788	SMU_788	RNA methyltransferase	0.632	0.03085
SMU_818	rpsU	30S ribosomal protein S21	0.648	0.03916
SMU_84	truA	tRNA pseudouridine synthase A	0.434	0.00795
SMU_865	SMU_865	30S ribosomal protein S16	0.559	0.00544
SMU_866	SMU_866	hypothetical protein SMU_866	0.586	0.00452
SMU_867	rimM	16S rRNA-processing protein RimM	0.612	0.00705
SMU_91	ropA	trigger factor	0.662	0.00001
SMU_910	gtfD	glucosyltransferase-S	0.311	0.00002

SMU_913	SMU_913	glutamate dehydrogenase	0.650	0.00000
SMU_919c	SMU_919c	ATPase, confers aluminum resistance	0.587	0.00011
SMU_924	tpx	lipid hydroperoxide peroxidase	0.515	0.00000
SMU_957	SMU_957	50S ribosomal protein L10	0.663	0.00001
SMU_960	rpoE	Probable DNA-directed RNA polymerase subunit delta	0.508	0.00000
SMU_960	rplL	50S ribosomal protein L7/L12	0.639	0.00096
SMU_989	asd	Aspartate-semialdehyde dehydrogenase	0.600	0.00282
SMU_99	fbaA	fructose-bisphosphate aldolase	0.598	0.00000

* Data are presented in ratio of the abundance of the *clpP* mutant (C) over the abundance of the wild-type (U).

Only proteins with a P value <0.05 and a reduction of >1.5-fold are presented.

Table S3d. Up-regulated proteins identified in the *clpP* mutant*

Gene ID	Gene Symbol	Description /putative function	Ratio: (C/U)	P-Value
SMU_1004	gtfB	Glucosyltransferase-I	5.288	0.00000
SMU_1009	SMU_1009	histidine kinase	1.994	0.00003
SMU_104	SMU_104	alpha-glucosidase	2.980	0.00067
SMU_1045c	SMU_1045c	inorganic polyphosphate/ATP-NAD kinase	2.064	0.00000
SMU_1050	krpS	ribose-phosphate pyrophosphokinase	1.810	0.00000
SMU_1077	pgm	phosphoglucomutase	1.776	0.00000
SMU_1098c	SMU_1098c	Oxidoreductase	2.674	0.01270
SMU_1168	SMU_1168	transcriptional regulator	1.895	0.01080
SMU_119	adh	Alcohol dehydrogenase	1.594	0.00048
SMU_1214	pyrC	dihydroorotase	1.621	0.00608
SMU_123	SMU_123	DNA polymerase III PolC	1.976	0.00596
SMU_125	SMU_125	hypothetical protein SMU_125	1.547	0.04140
SMU_1252	grk	Glycerate kinase	2.751	0.00003
SMU_1256c	SMU_1256c	hypothetical protein SMU_1256c	2.995	0.00000
SMU_1293c	SMU_1293c	hypothetical protein SMU_1293c	5.501	0.00011
SMU_1322	budC	Acetoin reductase	2.064	0.00021
SMU_1334	sfp	Phosphopantetheinyl transferase	4.694	0.02616
SMU_133c	SMU_133c	MDR permease	4.505	0.00055
SMU_137	mleS	malate dehydrogenase	29.370	0.00000
SMU_1389	pckA	hypothetical protein SMU_1389	2.286	0.00000
SMU_139	SMU_139	hypothetical protein SMU_139	30.437	0.00001
SMU_1396	gbpC	glucan-binding protein gbpC	1.721	0.00005
SMU_140	SMU_140	glutathione reductase	5.253	0.01644
SMU_1409c	SMU_1409c	transcriptional regulator	5.234	0.00171
SMU_1410	SMU_1410	reductase	6.071	0.03170
SMU_1412c	SMU_1412c	ABC transporter membrane protein subunit and ATP-binding	3.157	0.00000
SMU_1421	pdhC	branched-chain alpha-keto acid dehydrogenase E2 subunit	15.074	0.00000
SMU_1422	pdhB	Pyruvate dehydrogenase E1 component subunit beta	12.216	0.00058
SMU_1423	pdhA	Pyruvate dehydrogenase, TPP-dependent E1 component alpha	12.365	0.00008

SMU_1438c	SMU_1438c	Zn-dependent protease	1.850	0.00000
SMU_1454c	SMU_1454c	hypothetical protein SMU_1454c	2.317	0.01086
SMU_1455	mutX	Mutator protein, pyrophosphohydrolase	2.939	0.00959
SMU_1463c	SMU_1463c	hypothetical protein SMU_1463c	1.766	0.00092
SMU_1479	SMU_1479	hypothetical protein SMU_1479	2.303	0.00000
SMU_148	adhE	bifunctional acetaldehyde-CoA/alcohol dehydrogenase	3.222	0.00000
SMU_1499	rexA	exonuclease RexA	1.696	0.00060
SMU_1508c	SMU_1508c	coenzyme PQQ synthesis protein	5.368	0.00196
SMU_1509	rgg	transcriptional regulator	3.852	0.00015
SMU_1537	glgD	glycogen biosynthesis protein glgD	2.410	0.00001
SMU_1538	glgC	Glucose-1-phosphate adenylyltransferase;	2.031	0.00600
SMU_1557c	SMU_1557c	hypothetical protein SMU_1557c	1.879	0.00107
SMU_1561	trkB	potassium uptake system protein TrkB	2.545	0.00000
SMU_1562	trk	potassium uptake protein TrkA	2.912	0.00180
SMU_1563	pacL	Cation-transporting P-type ATPase PacL	3.236	0.00000
SMU_1568	malX	maltose ABC transporter substrate-binding protein	2.175	0.00000
SMU_1570	malG	maltose ABC transporter permease	2.222	0.00192
SMU_1571	SMU_1571	MsmK-like ABC transporter ATP-binding protein	2.258	0.01004
SMU_1576c	SMU_1576c	hypothetical protein SMU_1576c	3.803	0.00000
SMU_1577c	SMU_1577c	hypothetical protein SMU_1577c	2.091	0.02034
SMU_1616c	SMU_1616c	hypothetical protein SMU_1616c	7.786	0.00001
SMU_1622	pmsR	methionine sulfoxide reductase A	2.532	0.03662
SMU_1661c	SMU_1661c	signal peptidase II	1.830	0.00590
SMU_1662	holB	DNA polymerase III subunit delta'	1.656	0.00048
SMU_1679c	SMU_1679c	hypothetical protein SMU_1679c	3.374	0.00000
SMU_1681c	SMU_1681c	hypothetical protein SMU_1681c	1.942	0.02685
SMU_1693	hlyX	hemolysin	1.548	0.03346
SMU_1717c	SMU_1717c	putative deoxyribonucleotide triphosphate pyrophosphatase	1.863	0.03945
SMU_1723c	SMU_1723c	hypothetical protein SMU_1723c	5.315	0.00020
SMU_1733c	SMU_1733c	SNF helicase	2.181	0.00229
SMU_1746c	SMU_1746c	enoyl-CoA hydratase	1.647	0.00603
SMU_174c	SMU_174c	hypothetical protein SMU_174c	5.094	0.00000

SMU_1762c	SMU_1762c	hypothetical protein SMU_1762c	5.601	0.00652
SMU_179	SMU_179	hypothetical protein SMU_179	4.584	0.00017
SMU_180	SMU_180	Oxidoreductase	4.020	0.00000
SMU_1826	yfbQ	aminotransferase	1.502	0.00923
SMU_1833	recG	ATP-dependent DNA helicase, RecG	2.048	0.02491
SMU_1851	uvrA	excinuclease ABC subunit A	1.713	0.00059
SMU_1882c	SMU_1882c	hypothetical protein SMU_1882c	4.824	0.00003
SMU_1902c	SMU_1902c	hypothetical protein SMU_1902c	2.523	0.01165
SMU_1923c	SMU_1923c	transcriptional regulator NrdR	1.852	0.00000
SMU_193c	SMU_193c	hypothetical protein SMU_193c	8.308	0.03278
SMU_1954	groEL	molecular chaperone GroEL	2.775	0.00000
SMU_1955	groES	co-chaperonin GroES	2.596	0.00001
SMU_195c	SMU_195c	hypothetical protein SMU_195c	23.162	0.00497
SMU_1969c	SMU_1969c	transcriptional regulator	3.666	0.00022
SMU_198c	SMU_198c	conjugative transposon protein	15.071	0.00002
SMU_1996	ipk	4-diphosphocytidyl-2-C-methyl-D-erythritol kinase	5.243	0.02032
SMU_20	mreC	Cell shape-determining protein MreC	1.696	0.00007
SMU_2027	SMU_2027	transcriptional regulator	1.867	0.00001
SMU_2028	sacB	Beta-D-fructosyltransferase, Ftf	3.033	0.00000
SMU_2029	clpC	ATP-dependent Clp protease, ATP-binding subunit	2.118	0.00000
SMU_2030	ctsR	CtsR family transcriptional regulator	4.604	0.00003
SMU_205c	SMU_205c	hypothetical protein SMU_205c	28.756	0.00000
SMU_2074	nrdD	anaerobic ribonucleoside triphosphate reductase	1.907	0.00000
SMU_2084c	SMU_2084c	Transcriptional regulator Spx	6.089	0.00008
SMU_2089	hexB	DNA mismatch repair protein	1.737	0.00008
SMU_209c	SMU_209c	hypothetical protein SMU_209c	20.546	0.00000
SMU_2108c	SMU_2108c	transcriptional regulator	2.786	0.00001
SMU_210c	SMU_210c	hypothetical protein SMU_210c	22.862	0.00000
SMU_2115	SMU_2115	short-chain dehydrogenase	2.832	0.04733
SMU_2116	opuCa	Osmoprotectant amino acid ABC transporter ATP-binding protein	1.600	0.00070
SMU_2119	opuCd	osmoprotectant ABC transporter permease	2.113	0.00010
SMU_2127	SMU_2127	Succinate semialdehyde dehydrogenase	2.803	0.00000

SMU_235	SMU_235	hypothetical protein SMU_235	1.610	0.00026
SMU_245	mecA	adaptor protein	1.954	0.01743
SMU_267c	SMU_267c	bifunctional glutamate--cysteine ligase/glutathione synthetase	2.114	0.00000
SMU_307	pgi	glucose-6-phosphate isomerase	1.578	0.00003
SMU_323	gpsA	NAD(P)H-dependent glycerol-3-phosphate dehydrogenase	1.628	0.00006
SMU_385	SMU_385	glycoprotein endopeptidase	2.228	0.00000
SMU_386	SMU_386	ribosomal-protein-alanine acetyltransferase	2.300	0.00042
SMU_399	SMU_399	hypothetical protein SMU_399	2.484	0.00000
SMU_400	SMU_400	Secreted esterase	3.354	0.03773
SMU_402	pfl	pyruvate formate-lyase	4.233	0.00000
SMU_411c	SMU_411c	hypothetical protein SMU_411c	4.593	0.00059
SMU_412c	SMU_412c	cell-cycle regulation protein	1.587	0.00001
SMU_44	SMU_44	DNA mismatch repair protein	6.352	0.00000
SMU_440	SMU_440	hypothetical protein SMU_440	1.616	0.00004
SMU_46	SMU_46	hypothetical protein SMU_46	2.513	0.04435
SMU_47	SMU_47	hypothetical protein SMU_47	3.299	0.00254
SMU_526c	SMU_526c	transcriptional regulator	7.162	0.00080
SMU_527	SMU_527	hypothetical protein SMU_527	1.978	0.00008
SMU_562	clpE	ATP-dependent protease clpE	5.785	0.00000
SMU_570	feoB	Ferrous ion transport protein B	2.123	0.00092
SMU_580	SMU_580	exodeoxyribonuclease VII large subunit	1.682	0.00378
SMU_585	recN	DNA repair protein RecN	1.526	0.03516
SMU_633	SMU_633	thioesterase	6.303	0.00000
SMU_636	SMU_636	N-acetylglucosamine-6-phosphate isomerase	1.817	0.00074
SMU_678	SMU_678	Oxidoreductase	2.552	0.00002
SMU_692	SMU_692	hypothetical protein SMU_692	1.530	0.00514
SMU_743	SMU_743	hypothetical protein SMU_743	1.675	0.03391
SMU_746c	SMU_746c	hypothetical protein SMU_746c	1.864	0.00171
SMU_756	SMU_756	hypothetical protein SMU_756	2.194	0.01831
SMU_757	SMU_757	hypothetical protein SMU_757	2.707	0.00005
SMU_758c	SMU_758c	hypothetical protein SMU_758c	5.233	0.00004
SMU_769	SMU_769	hypothetical protein SMU_769	2.469	0.00049

SMU_80	hrcA	heat-inducible transcription repressor	5.016	0.00000
SMU_804	SMU_804	hypothetical protein SMU_804	2.253	0.00366
SMU_809	uvrB	excinuclease ABC subunit B	1.967	0.00236
SMU_821	dnaG	DNA primase	1.510	0.03192
SMU_832	SMU_832	hypothetical protein SMU_832	1.866	0.01273
SMU_837	SMU_837	reductase	1.684	0.00000
SMU_842	thiI	thiamine biosynthesis protein ThiI	1.501	0.00000
SMU_852	SMU_852	transcriptional regulator; CpsY-like protein	1.699	0.01041
SMU_854	SMU_854	pseudouridylate synthase	1.950	0.00000
SMU_882	msmK	multiple sugar-binding ABC transporter ATP-binding	2.928	0.00000
SMU_883	dexB	Dextran glucosidase DexB	2.757	0.04017
SMU_905	SMU_905	ABC transporter ATP-binding protein	1.504	0.00000
SMU_936	SMU_936	Amino acid ABC transporter ATP-binding protein	1.812	0.00738
SMU_937	SMU_937	mevalonate diphosphate decarboxylase	1.866	0.00002
SMU_943c	SMU_943c	Hydroxymethylglutaryl-CoA synthase	1.943	0.00000
SMU_949	clpX	ATP-dependent protease ATP-binding subunit ClpX	2.620	0.00000
SMU_985	bglA	beta-glucosidase	2.091	0.00000

* Data are presented in ratio of the abundance of the *clpP* mutant (C) over the abundance of the wild-type (U).

Only proteins with a P value <0.05 and a ratio >1.5 are presented.

Table S4. Proteins that were differentiately regulated from *clpP**

Gene ID	Gene Symbol	Description /putative function	Ratio (C/U)	Ratio (M/U)	P-Value (C/U)	P-Value (M/U)
SMU_2-21	rs3	30S ribosomal protein S3	1.014	1.507	0.99996	0.00262
SMU_96	rpoE	Probable DNA-directed RNA polymerase subunit delta	0.508	4.058	0.00000	0.00000
SMU_08	trcF	transcription-repair coupling factor	1.333	1.529	0.00552	0.00008
SMU_09	SMU_09	hypothetical protein SMU_09	0.800	0.591	0.67344	0.01406
SMU_1001	smf	DNA processing Smf protein	0.827	2.914	0.98712	0.01388
SMU_1004	gtfB	Glucosyltransferase-I	5.288	0.576	0.00000	0.00000
SMU_1005	gtfC	Glucosyltransferase-SI	1.116	0.646	0.51333	0.00004
SMU_1037c	SMU_1037c	histidine kinase	1.492	2.128	0.10492	0.00062
SMU_1041	SMU_1041	ABC transporter ATP-binding protein	0.730	2.308	0.22107	0.00004
SMU_1043c	SMU_1043c	Phosphotransacetylase	1.364	1.812	0.00000	0.00000
SMU_1052	SMU_1052	hypothetical protein SMU_1052	1.014	2.098	0.99999	0.00000
SMU_1054	SMU_1054	glutamine amidotransferase	0.816	1.590	0.49957	0.01040
SMU_1067c	SMU_1067c	ABC transporter permease	0.900	4.036	0.40834	0.00000
SMU_1068c	SMU_1068c	ABC transporter ATP-binding protein	1.089	4.157	0.83914	0.00000
SMU_1069c	SMU_1069c	hypothetical protein SMU_1069c	0.807	3.752	0.84871	0.00319
SMU_1070c	SMU_1070c	hypothetical protein SMU_1070c	0.819	3.452	0.96296	0.00354
SMU_1078c	SMU_1078c	ABC transporter ATP-binding protein	0.942	1.940	0.68077	0.00000
SMU_1083c	SMU_1083c	hypothetical protein SMU_1083c	0.563	1.830	0.00473	0.00132
SMU_1084	hemK	N5-glutamine S-adenosyl-L-methionine-dependent methyltransferase	0.980	2.362	1.00000	0.02277
SMU_1088	apbE	thiamine biosynthesis lipoprotein	1.495	1.542	0.05688	0.03197
SMU_1089	SMU_1089	hypothetical protein SMU_1089	1.425	1.607	0.00000	0.00000
SMU_1090	SMU_1090	hypothetical protein SMU_1090	1.261	1.870	0.27169	0.00014
SMU_1096	opuBa	choline transporter ABC transporter ATP-binding protein	1.353	1.562	0.00116	0.00001
SMU_1118c	SMU_1118c	ABC sugar transporter, permease	1.130	2.435	0.99797	0.02279
SMU_1132	pepN	aminopeptidase	0.927	0.556	0.98225	0.00031
SMU_114	SMU_114	PTS system fructose-specific transporter subunit IIBC	1.442	5.604	0.46673	0.00080
SMU_115	SMU_115	PTS system fructose-specific transporter subunit IIA	1.222	10.106	0.92808	0.00033
SMU_1169c	SMU_1169c	thioredoxin	0.800	3.802	0.86853	0.00033

SMU_1178c	SMU_1178c	Amino acid ABC transporter ATP-binding protein	1.110	2.002	0.99475	0.02875
SMU_1180	phnA	alkylphosphonate uptake protein	0.669	3.355	0.58675	0.00076
SMU_1182	mtlD	Mannitol-1-phosphate 5-dehydrogenase	1.024	1.789	0.99999	0.04564
SMU_1183	mtlA2	PTS system mannitol-specific transporter subunit IIA	0.905	5.073	0.83659	0.00000
SMU_1188	lepB	signal peptidase	1.211	1.567	0.00083	0.00000
SMU_119	adh	Alcohol dehydrogenase	1.594	0.611	0.00048	0.00040
SMU_1194	SMU_1194	ABC transporter ATP-binding protein	1.131	1.640	0.31896	0.00000
SMU_1204	parC	DNA topoisomerase IV subunit A	1.300	1.723	0.28458	0.00234
SMU_1210	parE	DNA topoisomerase IV subunit B	1.056	1.501	0.95803	0.00023
SMU_1214	pyrC	dihydroorotase	1.621	0.545	0.00608	0.00059
SMU_1247	eno	Enolase	1.007	0.539	0.99999	0.00000
SMU_1269	serB	phosphoserine phosphatase	0.510	2.399	0.03124	0.00643
SMU_1276c	SMU_1276c	septation ring formation regulator EzrA	1.138	1.575	0.52964	0.00014
SMU_129	adhC	branched-chain alpha-keto acid dehydrogenase E2 subunit	0.874	1.815	0.90164	0.00441
SMU_1302	adcA	surface adhesin	1.042	2.287	0.88437	0.00000
SMU_1306c	SMU_1306c	glmZ(sRNA)-inactivating NTPase	1.069	1.524	0.99329	0.01563
SMU_1307c	SMU_1307c	hypothetical protein SMU_1307c	1.043	1.911	0.99987	0.00079
SMU_1324	ftsX	cell-division protein FtsX	1.258	1.755	0.23976	0.00067
SMU_1325	ftsE	ABC transporter ATP-binding protein	1.260	1.792	0.00043	0.00000
SMU_1383	leuB	3-isopropylmalate dehydrogenase	0.936	0.539	0.80506	0.00000
SMU_1384	leuA	2-isopropylmalate synthase	1.064	0.522	0.89135	0.00000
SMU_1391c	SMU_1391c	hypothetical protein SMU_1391c	0.920	1.619	0.94834	0.00140
SMU_1396	gbpC	glucan-binding protein gbpC	1.721	0.325	0.00005	0.00000
SMU_1442c	SMU_1442c	hypothetical protein SMU_1442c	0.959	1.976	0.99974	0.00015
SMU_1443c	SMU_1443c	tributyrin esterase	1.421	0.630	0.01818	0.00227
SMU_1467	apt	Adenine phosphoribosyltransferase	0.941	1.663	0.87830	0.00000
SMU_1473c	SMU_1473c	Oxidoreductase	1.263	2.135	0.21335	0.00001
SMU_1487	SMU_1487	hypothetical protein SMU_1487	0.442	1.698	0.00006	0.00694
SMU_1513	smc	Chromosome segregation ATPase	1.425	1.603	0.16766	0.03113
SMU_1527	atpA	ATP synthase F0F1 subunit epsilon	0.886	1.997	0.82002	0.00014

SMU_1528	atpB	ATP synthase F0F1 subunit beta	0.875	2.082	0.00560	0.00000
SMU_1529	atpC	ATP synthase F0F1 subunit gamma	0.933	2.240	0.99753	0.00144
SMU_1530	atpD	ATP synthase F0F1 subunit alpha	0.892	2.174	0.42237	0.00000
SMU_1531	atpE	ATP synthase F0F1 subunit delta	0.878	2.390	0.70497	0.00000
SMU_1532	atpF	ATP synthase F0F1 subunit B	0.913	2.240	0.39764	0.00000
SMU_1533	atpG	ATP synthase F0F1 subunit A	0.923	2.031	0.99444	0.01222
SMU_155	pnpA	polynucleotide phosphorylase	0.836	0.588	0.32407	0.00008
SMU_158	cysS	cysteinyl-tRNA synthetase	0.731	0.544	0.38019	0.01126
SMU_1603	lguL	lactoylglutathione lyase	1.264	2.358	0.74214	0.00098
SMU_1613c	SMU_1613c	dephospho-CoA kinase	1.355	2.630	0.42267	0.00019
SMU_1632	pfs	5'-methylthioadenosine/S-adenosylhomocysteine nucleosidase	0.926	0.625	0.97326	0.00344
SMU_1641c	SMU_1641c	hypothetical protein SMU_1641c	0.548	2.849	0.00000	0.00000
SMU_1644c	SMU_1644c	hypothetical protein SMU_1644c	0.867	1.661	0.40477	0.00002
SMU_1665	livF	branched chain amino acid ABC transporter ATP-binding protein	0.648	1.647	0.00151	0.00026
SMU_1666	livG	branched chain amino acid ABC transporter ATP-binding protein	0.604	1.641	0.00216	0.00226
SMU_1678	SMU_1678	acyl-CoA thioesterase	1.164	1.775	0.03026	0.00000
SMU_1688	dltD	extramembranal protein, DltD protein	1.157	1.992	0.91060	0.00094
SMU_1712c	SMU_1712c	segregation and condensation protein B	0.861	1.860	0.90002	0.00344
SMU_1716c	SMU_1716c	hypothetical protein SMU_1716c	0.784	2.200	0.15343	0.00000
SMU_1771c	SMU_1771c	hypothetical protein SMU_1771c	1.099	3.176	0.99785	0.00011
SMU_1777	nrdI	flavoprotein NrdI	0.665	2.790	0.00011	0.00000
SMU_1788c	SMU_1788c	bacterocin transport accessory protein, Bta	0.402	3.729	0.00001	0.00000
SMU_1789c	SMU_1789c	hypothetical protein SMU_1789c	0.696	1.584	0.00505	0.00055
SMU_1799	nadD	nicotinic acid mononucleotide adenylyltransferase	0.865	0.523	0.21864	0.00000
SMU_1819	gatB	aspartyl/glutamyl-tRNA amidotransferase subunit B	0.768	0.651	0.01258	0.00010
SMU_1821c	SMU_1821c	aspartyl/glutamyl-tRNA amidotransferase subunit C	0.847	0.649	0.33531	0.00048
SMU_1823	pncA	Pyrazinamidase/nicotinamidase	0.826	0.368	0.79081	0.00009
SMU_1831	aspG	L-asparaginase	0.757	0.506	0.05937	0.00001
SMU_1834	alr	Alanine racemase	0.897	0.547	0.99306	0.01842
SMU_1836	aroG	phospho-2-dehydro-3-deoxyheptonate aldolase	0.672	0.449	0.00231	0.00000

SMU_1837	aroH	phospho-2-dehydro-3-deoxyheptonate aldolase	0.883	0.459	0.77778	0.00000
SMU_184	sloC	ABC transporter metal binding lipoprotein	0.526	0.534	0.00000	0.00000
SMU_1840	scrK	fructokinase	0.889	0.650	0.90949	0.03722
SMU_1841	scrA	PTS system sucrose-specific transporter subunit IIABC	1.079	2.069	0.99265	0.00044
SMU_1849	comEB	deoxycytidylate deaminase	1.375	0.576	0.25058	0.01155
SMU_1855	SMU_1855	hypothetical protein SMU_1855	1.411	1.645	0.00161	0.00002
SMU_1859	ssb	single-stranded DNA-binding protein	0.825	0.499	0.94086	0.04007
SMU_1860	rs6	30S ribosomal protein S6	0.603	0.515	0.00254	0.00025
SMU_1869	trxA	thioredoxin	0.975	2.534	0.99076	0.00000
SMU_1878	ptnC	PTS system mannose-specific transporter subunit IIC	0.722	1.673	0.01843	0.00015
SMU_1935c	SMU_1935c	hypothetical protein SMU_1935c	1.062	1.571	0.96576	0.00005
SMU_1939c	SMU_1939c	ABC transporter ATP-binding protein	0.931	1.702	0.99900	0.04661
SMU_1943	syl	leucyl-tRNA synthetase	0.659	0.460	0.00001	0.00000
SMU_1955	groES	co-chaperonin GroES	2.596	0.485	0.00001	0.00022
SMU_1957	SMU_1957	PTS system mannose-specific transporter subunit IID	1.295	2.946	0.77440	0.00063
SMU_1958c	SMU_1958c	PTS system mannose-specific transporter subunit IIC	1.381	3.613	0.04173	0.00000
SMU_1960c	SMU_1960c	PTS system mannose-specific transporter subunit IIB	0.641	3.714	0.00008	0.00000
SMU_1961c	SMU_1961c	PTS system sugar-specific transporter subunit IIA	0.512	1.603	0.00000	0.00000
SMU_1971c	SMU_1971c	thioredoxin	1.073	3.149	0.99995	0.03997
SMU_1975c	SMU_1975c	hypothetical protein SMU_1975c	1.123	1.817	0.98316	0.03582
SMU_2005	adk	adenylate kinase	0.841	2.652	0.33978	0.00000
SMU_2047	ptsG	PTS system glucose-specific transporter subunit IIABC	1.010	1.734	0.99977	0.00000
SMU_2079c	SMU_2079c	hypothetical protein SMU_2079c	0.647	1.558	0.00683	0.00424
SMU_2121c	SMU_2121c	hypothetical protein SMU_2121c	1.055	2.823	0.96110	0.00000
SMU_2129c	SMU_2129c	hypothetical protein SMU_2129c	0.968	2.110	1.00000	0.00000
SMU_2137c	SMU_2137c	hypothetical protein SMU_2137c	0.702	1.657	0.19138	0.00981
SMU_227c	SMU_227c	hypothetical protein SMU_227c	1.018	1.693	1.00000	0.00004
SMU_236c	SMU_236c	transcriptional regulator	0.792	0.544	0.78307	0.04992
SMU_245	mecA	adaptor protein	1.954	0.043	0.01743	0.00000
SMU_246	rgpG	glycosyl transferase N-acetylglucosaminyltransferase RgpG	1.302	0.136	0.30842	0.00000

SMU_250	nifU	nitrogen fixation-like protein, NifU	0.824	1.837	0.41694	0.00007
SMU_255	oppA	Oligopeptide ABC transporter substrate-binding protein OppA	0.639	1.602	0.00000	0.00000
SMU_270	sgaT	PTS system ascorbate-specific transporter subunit IIC	0.768	1.941	0.02107	0.00000
SMU_271	ptxB	PTS system transporter subunit IIB	0.649	2.941	0.00180	0.00000
SMU_298	SMU_298	hypothetical protein SMU_298	1.182	2.589	0.93243	0.00106
SMU_317	SMU_317	tetrahydrodipicolinate succinylase	0.686	0.553	0.00008	0.00000
SMU_325	SMU_325	Deoxyuridine 5'-triphosphate nucleotidohydrolase	0.732	0.451	0.01878	0.00000
SMU_334	SMU_334	Argininosuccinate synthase	0.678	1.711	0.00002	0.00000
SMU_338	SMU_338	RNA-binding protein	0.708	0.514	0.01363	0.00001
SMU_35	purN	Phosphoribosylglycinamide formyltransferase	0.688	5.325	0.75231	0.00024
SMU_352	SMU_352	ribulose-phosphate 3-epimerase	1.442	0.568	0.00001	0.00000
SMU_354	SMU_354	hypothetical protein SMU_354	1.407	1.612	0.26544	0.04306
SMU_384	SMU_384	hypothetical protein SMU_384	1.479	1.741	0.15920	0.02164
SMU_395	pepX	X-prolyl-dipeptidyl aminopeptidase	1.394	0.470	0.58349	0.01200
SMU_395	pepX	X-prolyl-dipeptidyl aminopeptidase	1.394	0.470	0.58349	0.01200
SMU_396	glpF	glycerol uptake facilitator protein	0.550	1.598	0.00039	0.00528
SMU_428	SMU_428	hypothetical protein SMU_428	1.368	2.110	0.62362	0.01194
SMU_438c	SMU_438c	(R)-2-hydroxyglutaryl-CoA dehydratase activator-like protein	0.804	2.353	0.94534	0.02850
SMU_455	pbp2x	Penicillin-binding protein 2X	0.780	1.830	0.00070	0.00000
SMU_458	SMU_458	ATP-dependent RNA helicase	0.867	1.536	0.56747	0.00100
SMU_459	SMU_459	ABC transporter amino acid binding protein	0.630	2.080	0.09151	0.00291
SMU_466	pepC	cysteine aminopeptidase	0.817	0.502	0.03015	0.00000
SMU_473	SMU_473	hypothetical protein SMU_473	1.221	1.565	0.54127	0.02019
SMU_474	luxS	S-ribosylhomocysteinase	1.031	0.405	1.00000	0.00014
SMU_494	SMU_494	fructose-6-phosphate aldolase	1.143	0.568	0.93678	0.00975
SMU_495	gldA	Glycerol dehydrogenase	1.000	0.499	0.99999	0.00013
SMU_503c	SMU_503c	hypothetical protein SMU_503c	1.432	7.628	0.32697	0.00001
SMU_508	SMU_508	hypothetical protein SMU_508	1.028	1.707	0.99936	0.00810
SMU_51	purK	phosphoribosylaminoimidazole carboxylase ATPase subunit	0.829	0.630	0.67820	0.02050
SMU_510c	SMU_510c	hypothetical protein SMU_510c	1.153	4.774	0.88774	0.00000

SMU_528c	SMU_528c	hypothetical protein SMU_528c	1.311	4.225	0.69490	0.00000
SMU_542	glk	Glucose kinase	0.714	0.577	0.00000	0.00000
SMU_549	murG	undecaprenyldiphospho-muramoylpentapeptide beta-N-acetylglucosaminyltransfe	1.337	1.662	0.34085	0.01790
SMU_557	divIVA	cell division protein DivIVA	0.849	1.747	0.64310	0.00125
SMU_558	SMU_558	isoleucyl-tRNA synthetase	0.686	0.509	0.20497	0.00452
SMU_561c	SMU_561c	hydrolase (MutT family)	0.909	3.583	0.99725	0.00004
SMU_567	SMU_567	Glutamine ABC transporter permease	0.881	2.145	0.98688	0.00378
SMU_568	SMU_568	Amino acid ABC transporter ATP-binding protein	0.781	1.800	0.07705	0.00002
SMU_573	SMU_573	hypothetical protein SMU_573	0.695	0.593	0.01350	0.00050
SMU_577	lytS	histidine kinase LytS	1.286	1.769	0.42552	0.01220
SMU_597	pbp2b	penicillin-binding protein 2B	1.115	2.777	0.95464	0.00000
SMU_609	SMU_609	40K cell wall protein	0.787	11.617	0.00006	0.00000
SMU_610	spaP	cell surface antigen SpaP	0.765	0.527	0.00005	0.00000
SMU_63c	SMU_63c	hypothetical protein SMU_63c	0.873	0.593	0.75760	0.00093
SMU_648	prtM	foldase PrsA	1.250	1.576	0.21245	0.00146
SMU_65	SMU_65	Protein tyrosine-phosphatase	0.707	1.818	0.00321	0.00000
SMU_668c	SMU_668c	ribonucleotide-diphosphate reductase subunit alpha	0.957	1.764	0.98894	0.00001
SMU_669c	SMU_669c	glutaredoxin	0.760	3.524	0.09857	0.00000
SMU_672	idh	isocitrate dehydrogenase	0.785	0.665	0.20833	0.00780
SMU_674	ptsH	phosphocarrier protein HPr	0.821	2.809	0.47735	0.00000
SMU_675	SMU_675	PTS system transporter protein EI	1.062	0.646	0.65951	0.00000
SMU_684	SMU_684	hypothetical protein SMU_684	0.811	0.606	0.59078	0.01303
SMU_685	SMU_685	hypothetical protein SMU_685	0.655	2.058	0.06995	0.00093
SMU_696	SMU_696	cytidylate kinase	1.436	1.701	0.07183	0.00330
SMU_718c	SMU_718c	hypothetical protein SMU_718c	1.013	0.586	1.00000	0.00000
SMU_723	SMU_723	Cadmium-transporting ATPase	0.960	1.509	0.98168	0.00004
SMU_73	SMU_73	hypothetical protein SMU_73	0.832	0.403	0.81066	0.00012
SMU_730	SMU_730	hypothetical protein SMU_730	0.801	0.604	0.23871	0.00193
SMU_759	SMU_759	protease	0.842	0.458	0.96931	0.02247
SMU_775c	SMU_775c	hypothetical protein SMU_775c	1.313	1.917	0.22587	0.00033

SMU_78	fruA	Exo-beta-D-fructosidase	0.850	1.372	0.00000	0.00000
SMU_785	aroK	shikimate kinase	1.045	3.205	0.99352	0.00000
SMU_790	SMU_790	hypothetical protein SMU_790	1.182	3.101	0.90632	0.00422
SMU_794	SMU_794	hypothetical protein SMU_794	1.263	3.213	0.80470	0.00009
SMU_796	SMU_796	hypothetical protein SMU_796	1.461	2.087	0.00023	0.00000
SMU_81	grpE	Heat shock protein GrpE	1.151	0.568	0.20604	0.00000
SMU_815	SMU_815	amino acid ABC transporter substrate-binding protein	0.764	1.572	0.04315	0.00039
SMU_819	mscL	large conductance mechanosensitive channel	0.894	1.865	0.85049	0.00013
SMU_824	rlmD	dTDP-4-keto-L-rhamnose reductase	1.021	1.614	0.99770	0.00000
SMU_826	rgpB	Rhamnosyltransferase	1.393	1.630	0.21476	0.02510
SMU_828	rgpD	polysaccharide ABC transporter ATP-binding protein	1.328	1.578	0.00005	0.00000
SMU_829	rgpE	glycosyltransferase	1.417	1.607	0.21599	0.03964
SMU_833	SMU_833	glycosyltransferase	1.294	1.613	0.20359	0.00235
SMU_838	gshR	glutathione reductase	0.810	0.510	0.79272	0.00626
SMU_849	SMU_849	50S ribosomal protein L27	0.947	0.507	0.99723	0.00020
SMU_862	SMU_862	permease	0.823	0.339	0.21859	0.00000
SMU_872	SMU_872	PTS system fructose-specific transporter subunit IIABC	0.906	1.552	0.96889	0.01811
SMU_891	hsdM	type I restriction-modification system DNA methylase	1.262	1.770	0.56690	0.00858
SMU_892	hsdS	type I restriction-modification system, specificity determinant	1.229	1.966	0.90675	0.03697
SMU_924	tpx	lipid hydroperoxide peroxidase	0.515	3.126	0.00000	0.00000
SMU_932	SMU_932	hypothetical protein SMU_932	1.000	3.464	1.00000	0.01650
SMU_950	SMU_950	GTP-binding protein YsxC	1.297	1.559	0.00001	0.00000
SMU_984	SMU_984	hypothetical protein SMU_984	0.634	6.845	0.58531	0.00003
SMU_990	dapA	dihydrodipicolinate synthase	0.728	0.627	0.04444	0.00179
SMU_991	SMU_991	ribonucleotide reductase	1.462	2.093	0.21353	0.00261
SMU_998	SMU_998	ABC transporter periplasmic ferrichrome-binding protein	0.780	1.700	0.09391	0.00011

* Data are presented in ratios of the abundance of the *mecA* (M) and *clpP* (C) mutant over the abundance of the wild-type UA159 (U).

Table S5: Proteins identified from affinity pull-down

Locus	Gene name	Description /putative function
SMU.02	dnaN	putative DNA polymerase III, beta subunit
SMU.82	dnaK	heat shock protein, DnaK (HSP-70)
SMU.104	yicI	putative alpha-glucosidase glycosyl hydrolase
SMU.123	polC	DNA polymerase III, alpha subunit
SMU.245	mecA	putative negative regulator of genetic competence MecA
SMU.247	sufC	putative ABC transporter, ATP-binding protein
SMU.251	sufB	conserved hypothetical protein possible ABC transporter, membrane
SMU.359	EFG fus tetO	translation elongation factor G (EF-G)
SMU.360	gap gapC	extracellular glyceraldehyde-3-phosphate dehydrogenase
SMU.546	bipA typA	putative GTP-binding protein
SMU.562	clpE	ATP-dependent protease ClpE
SMU.714	tuf tufA	translation elongation factor EF-Tu
SMU.754	hprK ptsK	HPr(serine) kinase/phosphatase
SMU.949	clpX	ATP-dependent protease Clp, ATPase subunit ClpX
SMU.1004	gtfB	glucosyltransferase-I
SMU.1005	gtfC	glucosyltransferase-SI
SMU.1054	guaA	putative glutamine amidotransferase
SMU.1187	glmS	glucosamine-fructose-6-phosphate aminotransferase
SMU.1340	bacA	putative surfactin synthetase
SMU.1341c	grs mycB	putative gramicidin S synthetase
SMU.1429	murC	putative UDP-N-acetylmuramyl tripeptide synthetase MurC
SMU.1461	rfbA rmlA	putative glucose-1-phosphate thymidyltransferase
SMU.1528	atpD	FoF1 membrane-bound proton-translocating ATPase, beta subunit
SMU.1533	atpB	FoF1 membrane-bound proton-translocating ATPase, a subunit
SMU.1591	ccpA regM	catabolite control protein A, CcpA
SMU.1635	gcaD glmU	putative UDP-N-acetylglucosamine pyrophosphorylase
SMU.1731	murC	putative UDP-N-acetyl muramate-alanine ligase
SMU.1954	groEL	putative chaperonin GroEL
SMU.1989	rpoC	DNA-dependent RNA polymerase, beta subunit
SMU.1990	rpoB	DNA-dependent RNA polymerase, beta subunit
SMU.2029	clpC	class III stress response-related ATP-dependent Clp protease
